# Supplementary material for: Comparative Transcriptome Analysis of Agrobacterium tumefaciens Reveals the Molecular Basis for the Recalcitrant Genetic Transformation of Camellia sinensis L
Source: Biomolecules. 2022 May 11;12(5):688. doi: 10.3390/biom12050688 (PMC9138961; doi:10.3390/biom12050688)
Supplement: Supplementary file 1 [file biomolecules-12-00688-s001.zip › biomolecules-1645209.pdf]

biomolecules

Article

Comparative Transcriptome Analysis of *Agrobacterium tumefaciens* Reveals the Molecular Basis for the Recalcitrant Genetic Transformation of *Camellia sinensis* L.

Ke Jin [1,2,†], Na Tian [1,2,†], Jorge Freire da Silva Ferreira [3], Devinder Sandhu [3], Lizheng Xiao [1], Meiyi Gu [1], Yiping Luo [2], Xiangqin Zhang [2], Guizhi Liu [2], Zhonghua Liu [1,2,*], Jianan Huang [1,2,*] and Shuoqian Liu [1,2,*]

1 Department of Tea Science, College of Horticulture, Hunan Agricultural University, Changsha 410128, China; emmaking0467@stu.hunau.edu.cn (K.J.); tianna5678@yahoo.com (N.T.); xiaolz1963@yahoo.com (L.X.); gumeiyi@outlook.com (M.G.)
2 Key Laboratory of Tea Science of Ministry of Education, Hunan Agricultural University, Changsha 410128, China; akalileu@outlook.com (Y.L.); zhangxiangqin00@outlook.com (X.Z.); lgz2169@outlook.com (G.L.)
3 United States Salinity Laboratory, United States Department of Agriculture, Agricultural Research Service, Riverside, CA 92507, USA; jorge.ferreira@usda.gov (J.F.d.S.F.); devinder.sandhu@usda.gov (D.S.)
* Correspondence: zhonghua-liu@hunau.edu.cn (Z.L.); jian7513@sina.com (J.H.); shuoqianliu@hunau.edu.cn (S.L.)
† These authors contributed equally to this work.

check for updates

Citation: Jin, K.; Tian, N.; da Silva Ferreira, J.F.; Sandhu, D.; Xiao, L.; Gu, M.; Luo, Y.; Zhang, X.; Liu, G.; Liu, Z.; et al. Comparative Transcriptome Analysis of *Agrobacterium tumefaciens* Reveals the Molecular Basis for the Recalcitrant Genetic Transformation of *Camellia sinensis* L. *Biomolecules* **2022**, *12*, 688. https://doi.org/10.3390/biom12050688

Academic Editor: Kyoungwhan Back

Received: 4 March 2022
Accepted: 9 May 2022
Published: 11 May 2022

Publisher's Note: MDPI stays neutral with regard to jurisdictional claims in published maps and institutional affiliations.

Copyright: © 2022 by the authors. Licensee MDPI, Basel, Switzerland. This article is an open access article distributed under the terms and conditions of the Creative Commons Attribution (CC BY) license (https://creativecommons.org/licenses/by/4.0/).

Abstract: Tea (*Camellia sinensis* L.), an important economic crop, is recalcitrant to *Agrobacterium*-mediated transformation (AMT), which has seriously hindered the progress of molecular research on this species. The mechanisms leading to low efficiency of AMT in tea plants, related to the morphology, growth, and gene expression of *Agrobacterium tumefaciens* during tea-leaf explant infection, were compared to AMT of *Nicotiana benthamiana* leaves in the present work. Scanning electron microscopy (SEM) images showed that tea leaves induced significant morphological aberrations on bacterial cells and affected pathogen–plant attachment, the initial step of a successful AMT. RNA sequencing and transcriptomic analysis on *Agrobacterium* at 0, 3 and 4 days after leaf post-inoculation resulted in 762, 1923 and 1656 differentially expressed genes (DEGs) between the tea group and the tobacco group, respectively. The expressions of genes involved in bacterial fundamental metabolic processes, ATP-binding cassette (ABC) transporters, two-component systems (TCSs), secretion systems, and quorum sensing (QS) systems were severely affected in response to the tea-leaf phylloplane. Collectively, these results suggest that compounds in tea leaves, especially gamma-aminobutyrate (GABA) and catechins, interfered with plant–pathogen attachment, essential minerals (iron and potassium) acquisition, and quorum quenching (QQ) induction, which may have been major contributing factors to hinder AMT efficiency of the tea plant.

Keywords: *Camellia sinensis*; *Agrobacterium tumefaciens*; genetic transformation; transcriptomic analysis; tea leaf; AMT

1. Introduction

Tea (*Camellia sinensis* (L.) O. Kuntze) is a widely cultivated and commercially valuable crop because its leaves can be processed into beverages or food additives [1]. Its perennial and woody nature, long growth cycle, and low success rates for hand pollination have restricted the improvement of tea cultivars through conventional cross breeding [2]. Therefore, novel breeding technology, such as molecular breeding, is urgently needed to enhance breeding efficiency in the tea crop.

In plant molecular breeding, the *Agrobacterium*-mediated transformation (AMT) is the most effective tool to produce novel cultivar with desired traits. AMT has successfully improved staple crops, such as tomato [3], maize, and soybean [4], through its simple

procedure and high transformation rates. Moreover, AMT is a fundamental plant genetic engineering approach for gene function elucidation, validation, and genome editing.

However, tea plants are recalcitrant to AMT, which has seriously hindered the progress of molecular research on this species. Currently, only three successful cases of AMT have been reported in tea plants [5–7], all of which used cotyledon-induced somatic embryos as explants. No successful AMT based on tea-leaf tissue as explants, which maintains better genetic traits [8], has been reported. In our previous research (unpublished), we observed no fluorescence when *Agrobacterium* containing a GFP or LUC over-expression cassette was injected into tea leaves or in callus generated from tea leaves, while strong fluorescence was observed in tobacco leaves, confirming that the AMT in tea is still a big challenge. Although the whole genome sequence of tea plants was released over five years ago [9], the deep functional analysis of genes in tea plants has not been performed, due to the lack of a stable AMT system in tea. Great efforts have been made to optimize the tea AMT system, including the use of different bacterial strains [10], different types of explants [5,6,8], and different co-culture conditions [11–13]. Unfortunately, no significant progress has been made. Therefore, the advancement of tea genetic improvement relies on unveiling the reasons for the recalcitrant genetic transformation of *Camellia sinensis* through AMT.

Normally, the agrobacterial pathogenic process, critical to a successful AMT, is comprised of the following six steps: (1) plant-derived signal reception and bacterial chemotaxis, (2) virulence induction, (3) pathogen–plant attachment (reversible and irreversible), (4) transfer DNA (T-DNA) generation and transfer into plant cells, and (5) T-DNA trafficking and insertion into host cells (Figure 1). *Agrobacterium* has an advanced chemotactic signaling mechanism with a VirA/VirG two-component signal transduction system (TCS) [14]. When the membrane-spanning sensor protein, VirA, recognizes the wound-triggered plant signals, it phosphorylates the sequence-specific DNA-binding protein VirG, which in turn regulates the expression of the other *vir* genes required for the infection process. Thus, plant–pathogen interaction is required for pathogenicity. The virulent attachment process occurs stepwise with an initial reversible step, followed by an irreversible attachment [15]. Bacterial cells swim towards plant wounds attracted by plant-derived signals, with this process relying on effective bacterial chemotaxis [16]. Once the bacterium arrives at the host cell surface, reversible attachment is established by several types of proteins named pilin (fibrous proteins found in bacterial pilus structures) and adhesins [17]. Afterward, unipolar polysaccharides (UPP), secreted by *Agrobacterium* cells, aggregate newborn cells to form small bacterial colonies [18]. In the colony-forming process, *Agrobacterium* builds a biofilm to facilitate irreversible attachment [19]. This biofilm consists of exopolysaccharides (EPS), exogenous DNA (eDNA), and proteins.

In the meanwhile, T-complexes are generated inside the bacterial cell. The T-complex consists of single-stranded T-DNA and various Vir proteins. Those Vir proteins (VirD1, D2, C1, C2, etc.) help generate and protect the T-DNA and direct it to the host-cell nuclei [14,15]. The T-complexes enter the plant cell through a type-IV secretion system (T4SS), involving VirD4 and VirB proteins [15]. Another well-studied bacterial secretion system is the type-VI secretion system (T6SS). T6SS is recognized as a nanomachine used to colonize around the host wound and to inject effectors or toxins through a bacteriophage tail-like structure [20]. T6SS attacks both eukaryotic and prokaryotic cells [20] and is triggered by unfavorable conditions, such as carbon starvation [21] and reactive oxygen species (ROS) [22].

Quorum sensing (QS) is a piece of bacterial machinery that performs cell-cell communication through autoinducers [23,24], mainly regulating horizontal gene transfer [25] and pathogenesis [26]. QS begins with the binding of the TraR protein to the signal molecule 3-oxo-octanoylhomoserine lactone (OC8-HSL) [27]. Afterwards, the TraR-OC8-HSL complex induces the transcription of genes encoding DNA transfer and replication [28]. Contrary to the QS, if the bacteria sense a non-conducive environment for growth, quorum quenching (QQ) can disturb the QS pathway by targeting QS signals [29]. The TraM protein, a QQ regulator, binds to the TraR protein, occupying the binding site of OC8-HSL [28], thus, disrupting the QS pathway. Plants have developed defense systems to modulate bacterial

QS systems by releasing inhibitors [30]. For instance, plant-derived gamma-aminobutyrate (GABA) is a signal jammer for the QS pathway [31]. It takes advantage of the ATP binding cassette (ABC) transporter, Bra, and a periplasmic binding protein, Atu2422, to enter *Agrobacterium* [31]. GABA is transformed into succinic semialdehyde (SSA) inside bacterial cells, which inhibits BlcR, a transcriptional repressor of the *blcC* gene. Thus, when *blcC* is up-regulated, it encodes BlcC lactonase, which cleaves OC8-HSL, thus, strengthening the QQ process [28,32].

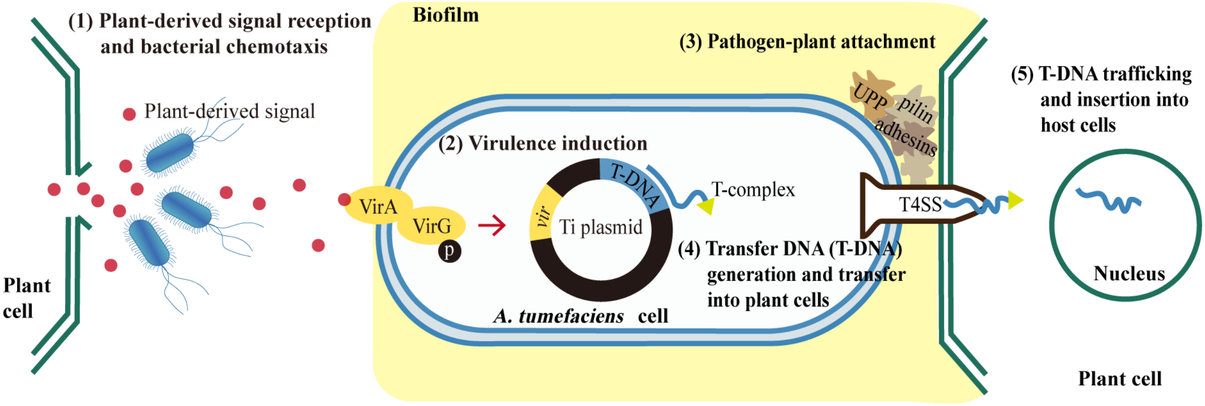

Figure 1. The process of *Agrobacterium*-mediated transformation in plants. *Agrobacteria* sense the plant-derived signals and swim towards the wounded plant cells. VirA protein on the membrane of *A. tumefaciens* cell recognizes the wound-triggered plant signals and phosphorylates the sequence-specific DNA-binding protein VirG, which in turn regulates the expression of other *vir* genes required for the infection process. Pathogen–plant attachment is established through the production of pilin, adhesins, unipolar polysaccharides (UPP) and the formation of biofilm. T-complex consists of T-DNA and various Vir proteins and it enter plant cells through a type IV secretion system (T4SS). Finally, T-DNA is transferred into the plant nucleus and inserted into the plant genome.

Some impact factors that affect the transformation efficiency of *Agrobacterium* have been recognized. For instance, the plant-derived phenolic acetosyringone (AS) is commonly added to induce *vir* genes before transformation [33] because it is recognized by the VirA/VirG two-component system, located in the Ti plasmid, as a host-specific signal, and activates *vir* gene expression. Surprisingly, while the induction of VirA/VirG proteins is the most popular targeted step, modifications of the subsequent steps are rarely explored [15]. Besides, numerous functional genes have not been identified or studied, due to the complex prokaryotic regulatory networks and multifaceted, dynamic host–pathogen interactions [34].

To understand the molecular mechanisms involved in the low efficiency of AMT in tea plants, we analyzed the gene expression profiling in *Agrobacterium* co-cultivated with tea leaves, using tobacco leaves as a control due to their high (95%) AMT efficiency [35]. At the same time, scanning electron microscopy (SEM) was performed to evaluate the effect of the tea-leaf surface environment (phylloplane) on agrobacterial growth and attachment to host cells. Understanding the biological mechanisms that result in low AMT efficiency in tea will help enhance transformation techniques for improved AMT efficiency, which is critical for future tea breeding.

2. Materials and Methods

2.1. Materials

The plant materials used in this study were all from aseptic seedlings. The seedlings of *C. sinensis* cultivar Bixiangzao were cultured in 1/2 MS (Murashige and Skoog) medium (pH 5.8), and *N. benthamiana* seedlings were cultured in standard MS medium, under a 16 h/8 h light/dark cycle, at 25 °C and 74% humidity. *N. benthamiana* groups (ND0, ND1,

ND3, ND4) were defined as the control groups. The *Agrobacterium* strain used was the GV3101 (preserved in our laboratory), which contains the pMKV060 plasmid. PMKV060 was donated by Daniel Voytas [36] (Addgene plasmid #133315; (accessed on 16 January 2020) http://n2t.net/addgene:133315; RRID: Addgene_133315).

2.2. Bacterial Culture

GV3101 (previously transformed) was inoculated in liquid LB medium (LB; 50 mg·L^{-1} rifampicin; 50 mg·L^{-1} kanamycin; 25 mg·L^{-1} gentamicin) in the dark overnight (28 °C, 200 rpm). The bacterial cells were collected by centrifugation at 6500 rpm. To avoid the additives used to induce *Agrobacterium* virulence, we only use 1/2 × MS liquid medium to resuspend the collected cells and measure their OD_{600} = 0.6 (optical density at 600 nm).

2.3. Agrobacterium-Mediated Transformation (AMT)

Tea leaves (the third/fourth-youngest leaf) from aseptic seedlings were cut into small discs (0.5 cm × 0.5 cm). The leaf discs were then soaked in bacterial culture (described above) for 20 min. The infection of tobacco leaves was performed in the same way as tea leaves, as described above.

2.4. Scanning Electron Microscopy Observations

After infection, the drained leaf discs were plated into 1/2 × MS solid medium and co-cultured for 30 min (D0), 24 h (D1), 72 h (D3), and 96 h (D4). The co-culture was carried out in the dark for the first two days and shifted to a 16 h/8 h light/dark cycle at 28 °C. Each treatment was conducted in triplicates. The collected discs at each time-point were washed with a 0.01 M PBS buffer (7.2–7.4 pH) and post-fixed by a fixative solution (Servicebio, Wuhan, China), and rinsed three times in 0.1 M PBS buffer for 15 min each time. Then, the discs were fixed with 1% osmium tetroxide (OsO_4) in 0.1 M PBS (7.4 pH) for 30 min. After this, the discs were washed in 0.1 M PBS (7.4 pH) 3 times, 15 min each time. The leaf discs were dehydrated by sequential aqueous solutions of 30%, 50%, 70%, 80%, 90%, 95%, and 100% ethanol for 15 min each, followed by a 15-min treatment with isoamyl acetate. A critical-point dryer was used to dry the samples. The dried samples were affixed to metallic stubs, sputter-coated with gold, taken to a SEM (SU8100, Hitachi, Japan), and photographed under the following analytical conditions: EHT = 3.0 KV, working distance = 12.4 mm; signal = SE(L).

2.5. Transcriptomic Sequencing and Analysis

After transformation, the leaf discs were dried in a flow hood to remove residue water; then, discs were cultured on liquid MS medium for 30 min (D0, in the dark), 72 h (D3, first two days in the dark following one day in the light), and 96 h (D4, first two days in the dark following two days in the light). Then, the bacterial cells were harvested by centrifugation at 7500 rpm for 5 min at 4 °C. The pellet was washed with sterile water and harvested by centrifugation at 7500 rpm for 10 min at 4 °C, then immediately frozen in liquid nitrogen, and stored at −80 °C. The total RNA of *Agrobacterium* was extracted using TRIzol reagent (Invitrogen, Waltham, MA, USA). The integration and quality of the extracted total RNA were examined by the RNA Nano 6000 Assay Kit of the Bioanalyzer 2100 system (Agilent Technologies, Santa Clara, CA, USA). Probes were used to remove rRNA and purify mRNA from the total RNA. Then, fragmentation, synthesis of the first- and second-strand complementary DNA (cDNA), adenylation of 3′ DNA ends, degradation of the second strand of cDNA containing U bases, purification of the library fragments, PCR reaction, and product purification were carried out to construct a cDNA library. The clustering was carried out with a cBot Cluster Generation System using the TruSeq PE Cluster Kit v3-cBot-HS (Illumina, San Diego, CA, USA), and sequencing was performed on the Illumina Novaseq platform.

After the quality control of raw data was established, clean reads were aligned to the reference genome of *Agrobacterium tumefaciens*, plus the plasmid pMKV060, using Bowtie2-

2.2.3. Gene annotation was performed on Rockhopper. The mapped read numbers of each gene were counted using HTSeq v0.6.1, and the expected number of fragments per kilobase of transcript sequence per million base pairs sequenced (FPKM) was calculated. After this, differential expression analysis of three comparisons (CD0 vs. ND0; CD3 vs. ND3; CD4 vs. ND4) was performed using a DESeq R package (1.18.0) by the standard of Benjamini and Hochberg's approach [37], adjusted p-value (P_{adj}) < 0.05. Gene Ontology (GO) enrichment analysis and KEGG (Kyoto Encyclopedia of Genes and Genomes) enrichment analysis of DEGs (Differentially Expressed Genes, P_{adj} < 0.05) were implemented by the GOseq R package and KOBAS 2.0 [38].

2.6. Quantitative Reverse Transcription-PCR (qRT-PCR) Verification

The bacterial total RNA was extracted by the Bacterial RNA Kit (Omega Bio-Tek, Norcross, GA, USA). cDNA was synthesized using the PrimeScript^{TM} RT Reagent Kit (TaKaRa, Dalian, China) and was applied to the qRT-PCR reaction with TB Green Premix Ex Taq^{TM} II (TaKaRa, Dalian, China). The reaction was performed in biological triplicates, with three technical replications on ABI QuantStudio™ III (Applied Biosystems, Foster City, CA, USA). The primers used in this study are presented in Table S1. The relative expression values were normalized with three housekeeping genes, *gyrB* (*atu0012*, GenBank accession number AE007869.2), *dnaC* (*atu1084*, GenBank accession number AE007869.2), and *atu8171* (GenBank accession number AE007869.2). Pearson correlation coefficient analyses of the RNA-seq and qRT-PCR data sets were performed.

2.7. Statistical Analysis

Statistical analyses were conducted using the SPSS software; pictures were plotted using the GraphPad, TB (Toolbox for Biologists) tools software. All the statistical comparisons were performed using one-way ANOVA and Student's *t*-test; p values $\leq$ 0.05 were considered significant.

3. Results

3.1. Growth and Morphological Changes in Agrobacterium during Transformation

The growth and attachment of *Agrobacterium* cells co-cultivated on explants from tobacco and tea plants after infection were observed using an SEM (Figure 2). Significant morphological aberrations of the bacterial cells were detected on the tea-leaf discs, compared to tobacco leaf discs. On the first day of co-cultivation with tobacco leaf discs, *Agrobacterium* cells tended to locate on the abaxial surface of discs, rather than on their cross-section (Figures 2A and S1A). The *Agrobacterium* cells on tea leaves were attached to the cross-section, especially on vascular bundles (Supplementary Figure S2B). At the early stage of co-cultivation, only a few bacterial clusters were observed on the tobacco leaves, and the morphology of the cells was similar to those attached to the tea leaves (Figure 2A,B). After one day of co-cultivation, the total number of *Agrobacterium* cells was large, and the length of single bacterial cells was higher on the tobacco leaves than on tea leaves (Figure 2C,D). Most of the bacterial cells on the tea leaves were minicells, being short and swollen (Figure 2D). At the early stage of co-cultivation, *Agrobacterium* cells in both tea and tobacco groups were branched with multiple growth poles (Figure 2B,E). However, the sizes of the branched bacterial cells on the tea leaves were much larger than those on the tobacco leaves and appeared to have several constriction sites (Figure 2B), probably due to defects in cell division. In the tobacco groups, the bacterial clusters adhered to the plant cells in a polar orientation (Figure 2A,C,E,G). However, in the tea groups, the bacterial clusters were wrapped in cellulose, scattered across the surface of discs (Figure 2H). Furthermore, both polar and lateral attachments were observed in the tea groups (Figure 2B,D,F,H).

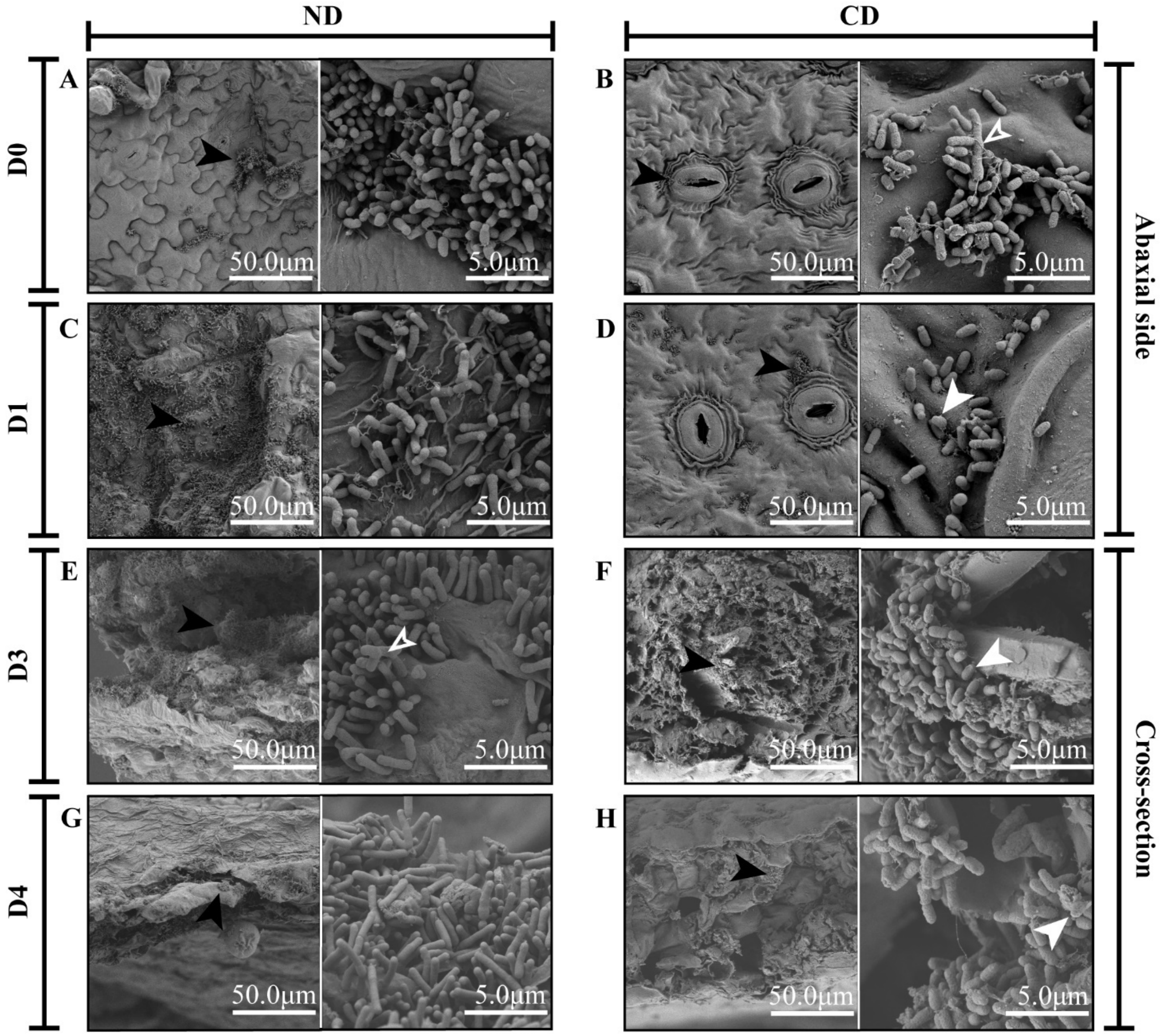

Figure 2. Scanning electron microscopy (SEM) images of *Agrobacterium* GV3101 on tobacco leaves (**A**,**C**,**E**,**G**) and tea leaves (**B**,**D**,**F**,**H**) at different time-points. (**A**,**B**) 30-min co-cultivation. (**C**,**D**) 24-h co-cultivation. (**E**,**F**) 72-h co-cultivation. (**G**,**H**) 96-h co-cultivation. Solid black arrowheads point to bacteria clusters; solid white arrowheads to minicells; and hollow white arrowheads to branched/swollen cells.

3.2. Transcriptomic Analysis of Agrobacterium during Genetic Transformation

This study compared samples from the following three time-points: CD0 (*Agrobacterium* cells co-cultivated with phylloplane of *C. sinensis* (tea) for 30 min) vs. ND0 (*Agrobacterium* cells co-cultivated with phylloplane of *N. benthamiana* (tobacco) for 30 min); CD3 (*Agrobacterium* cells co-cultivated with phylloplane of *C. sinensis* for 3 d) vs. ND3 (*Agrobacterium* cells co-cultivated with phylloplane of *N. benthamiana* for 3 d); CD4 (*Agrobacterium* cells co-cultivated with phylloplane of *C. sinensis* for 4 d) vs. ND4 (*Agrobacterium* cells co-cultivated with phylloplane of *N. benthamiana* for 4 d). A total of 18 transcriptomes of *Agrobacterium* from the tea and tobacco groups with co-cultivation for 30 min (CD0 or ND0), 3 d (CD3 or ND3), and 4 d (CD4 or ND4) were established. All the treatments were performed in triplicate (for a total of 18 transcriptomic samples). Regrettably, one biological replicate of the sample ND3 from the tobacco group showed a low mapping rate (about 78%) of raw reads against the reference genome of *A. tumefaciens str. C58* and the plasmid pMKV060. A 78% mapping rate led the sample to be disqualified for further analysis, so we

eliminated the sample and performed further analyses with the remaining two biological replicates of ND3. Thus, the total clean reads for each sample ranged from 6,584,706 to 8,962,786 in the remaining 17 samples, with an average mapping rate of 97.15% to the reference genome (Table S2). This dataset has been deposited in NCBI with BioProject number PRJNA764576.

A total of 5359 *Agrobacterium* genes were mapped on the reference genome in three comparisons. A total of 762, 1923, and 1656 differentially expressed genes (DEGs) were found in the tea groups compared to the tobacco groups at the same time-points on day 0, day 3, and day 4, respectively. Among those DEGs, 139 *Agrobacterium* genes were down-regulated, and 114 were up-regulated in the *Agrobacterium*-tea groups compared to *Agrobacterium*-tobacco groups, at all three-time points (Figure S2A). Among the 139 down-regulated genes described above, 101 were localized on the circular chromosome and none on the pMKV060 plasmid (Figure S2B). Of the 114 up-regulated genes, the number of those mapped on the linear chromosome and that on the circular chromosome were close (45 and 50), and 1, 7, 11 genes mapped on plasmid pMKV060, tumor-inducing plasmid (pTi), and At plasmid, respectively (Figure S2B).

3.3. Gene Ontology Enrichment Analysis

Gene Ontology (GO) enrichment analyses against the DEGs in each comparison were performed to identify their biological function. All the significantly enriched GO terms on the first day of co-cultivation (day 0) belonged to the biological process (BP) category (Figure S3). On day 3, 38 BP terms, 15 cellular-component (CC) terms, and 2 molecular-function (MF) terms were identified (Figure S3). On day 4, 16 BP terms, 14 CC terms, and 2 MF terms were significantly enriched (Figure S3). On the first day of co-cultivation, the most significantly enriched terms in BP were related to the ATP metabolic process (GO:0046034), purine nucleoside triphosphate metabolic process (GO:0009144), and ribonucleoside triphosphate metabolic process (GO:0009199) (Table S3). On both days 3 and 4, the top three enriched terms in the CC category were cytoplasmic part (GO:0044444), intracellular ribonucleoprotein complex (GO:0030529), and ribonucleoprotein complex (GO:1990904), while the two terms, structural constituent of ribosome (GO:0003735) and structural molecule activity (GO:0005198) in the MF category were significantly enriched (Table S3). On day 3, peptide biosynthetic process (GO:0043043), translation (GO:0006412), and peptide metabolic process (GO:0006518) were significantly enriched and assigned in the BP, whereas ATP metabolic process (GO:0046034), purine nucleoside monophosphate metabolic process (GO:0009126), and ribonucleoside monophosphate metabolic process (GO:0009161) were significantly enriched on day 4 (Table S3).

3.4. Kyoto Encyclopedia of Genes and Genomes (KEGG) Enrichment Analysis

KEGG pathway enrichment analyses (Table S4) were performed to characterize the bacterial pathways affected by tea as a host compared to tobacco during plant transformation. The top 30 pathways among 81 enriched pathways were presented in circle plots (Figure S4). Out of these 81 pathways, 65 pathways were involved in the metabolism category, 8 in the genetic information processing, 3 in cellular processes, 3 in the environmental information processing, and 2 in drug resistance (antimicrobial). The three most enriched pathways were oxidative phosphorylation (ko00190), sulfur metabolism (ko00920), and citrate cycle (ko00020) on day 0, ribosome (ko03010), oxidative phosphorylation (ko00190), and 2-oxocarboxylic acid metabolism (ko01210) on day 3, and oxidative phosphorylation (ko00190), ribosome (ko03010), and citrate cycle (ko00020) on day 4 (Figure S4 and Table S4). Additionally, all the DEGs in these top enriched pathways were down-regulated in the tea groups, compared to the tobacco groups.

Under the genetic information processing category, the top three enriched pathways were classified into two subcategories that were 'translation' (ribosome, ko03010; aminoacyl-tRNA biosynthesis, ko00970) and 'folding, sorting, and degradation' (RNA degradation, ko03018). In ko03010, there were 13 DEGs enriched on day 0, 53 DEGs on day

3, and 37 DEGs on day 4; all were down-regulated (Figure S4 and Table S4). In ko00970, there were 9 DEGs down-regulated on day 0; 2 DEGs were up-regulated, and 19 DEGs were repressed on day 3; 1 gene was up-regulated, and 6 were down-regulated on day 4 (Figure S4 and Table S4). In pathway ko03018, there were three DEGs enriched on day 0, two of them were down-regulated and the other one was up-regulated; on day 3, one DEG (*recQ*) was up-regulated and nine were down-regulated; on day 4, all nine DEGs were down-regulated (Figure S4 and Table S4).

Compared with the tobacco groups, six enriched pathways involved in the AMT process were found in *Agrobacterium* in the tea groups, including TCS (ko02020), ABC transporters (ko02010), bacterial secretion system (ko03070), flagellar assembly (ko02040), QS (ko02024), and bacterial chemotaxis (ko02030) (Figure S4, Table S4). ko02020, ko02010, and ko03070 belong to the category environmental information processing, while the remaining three pathways belong to the category cellular processes. In the pathway of TCS (ko02020), there were 22 DEGs enriched on day 0, 57 on day 3, and 44 on day 4 (Figure S4, Table S4). For the ABC transporter pathway (ko02010), there were 72, 137, 135 enriched DEGs on days 0, 3, and 4, respectively (Figure S4, Table S4). Four DEGs annotated for the bacterial secretion system (ko03070) were found on day 0, 24 on day 3, and fifteen on day 4 (Figure S4, Table S4). In the flagellar assembly pathway (ko02040), on day 0, there were 9 down-regulated genes and 1 up-regulated gene; on day 3, all 17 DEGs enriched in the pathway were suppressed; on day 4, 2 genes were up-regulated, and the other 19 DEGs were down-regulated (Figure S4, Table S4). In the QS system, 26 DEGs consisted of 16 up-regulated genes and 10 down-regulated genes on day 0; 55 up-regulated and 39 down-regulated genes on day 3; and 27 up-regulated and 36 down-regulated genes on day 4 (Figure S4, Table S4). In the bacterial chemotaxis pathway, all the DEGs enriched were down-regulated on day 0; 9 DEGs were up-regulated, while 15 were down-regulated on day 3; and 8 DEGs were up-regulated and 10 were down-regulated on day 4. The detailed regulations of the genes enriched in the six pathways mentioned above are described in the following section.

3.5. Transcriptional Changes of Genes Related to Agrobacterium-Mediated Transformation (AMT)

3.5.1. Expression Pattern Analysis of Genes Related to Environmental Information Processing

As mentioned previously, there were three pathways classified to the environmental information processing category, according to the KEGG enrichment results. In the TCS pathway (ko02020), there were three gene families (chemotaxis family, cell cycle family, ompR family) responding to tea leaves (Table S4). In the chemotaxis family, the *mcp* genes, *cheA*, *cheW1*, and *cheW2* in the tea groups were down-regulated at all three time-points (days 0, 3, and 4); *cheY* (atu0516, atu0520) was down-regulated on days 0 and 3 (Figure 3A). In the cell cycle family, the expression of *pleD* was increased by 1.5-fold and *ctrA* was decreased by 0.62-fold in the tea group, compared to the tobacco group on day 0; *ctrA* and *divk* were down-regulated to 0.35-fold and 0.41-fold, respectively, on day 3 (Figure 3A and Table S5). In the ompR family, of the *kdp* genes, *kdpB*, *kdpC*, *kdpD* were up-regulated on day 3 and *kdpB* and *kdpE* were up-regulated on day 4 in the tea group.

In the ABC transporter pathway (ko02010) of *Agrobacterium*, the transport systems of alkanesulfonate (*ssuA* = *atu1884*), mannopine (*attC* = *atu5129*), alpha-glucoside (*aglF* = *atu0592*, *aglG* = *atu0593*, *aglK* = *atu0595*), and dipeptide (*dppA* = *atu4113*) were all down-regulated during the whole process of co-cultivation in the tea groups (Figure 3B). Meanwhile, the transport systems of iron (*fbpA* = *atu0407*, *afuA* = *atu4784*, *afuB* = *atu4785*, *afuC* = *atu4786* and others), maltose (*atu0391*, *atu4559*, *atu4450*), oligogalacturonide (*atu3130*, *atu3132*), glucose (*atu3351*, *atu3352*), rhamnose (*atu3487–3490*), sn-glycerol 3-phosphate (*ugpC* = *atu3099*/*atu3188*), branched-chain amino acid (*livH* = *atu4518*, *livG* = *atu4516*, *livM* = *atu4517*, and others) and urea (*atu5531–5533*) were all up-regulated during the whole process of co-cultivation in the tea groups, compared with the tobacco groups at each time-point (Figure 3B and Table S5). *NocP* (*atu6028*) and *nocQ* (*atu6026*), both encoding the nopaline transport system,

were up-regulated on day 0 in the tea group, while *nocP* was also highly up-regulated (by 4.8-fold) on day 4 in the tea group, compared to the tobacco group (Figure 3B and Table S5).

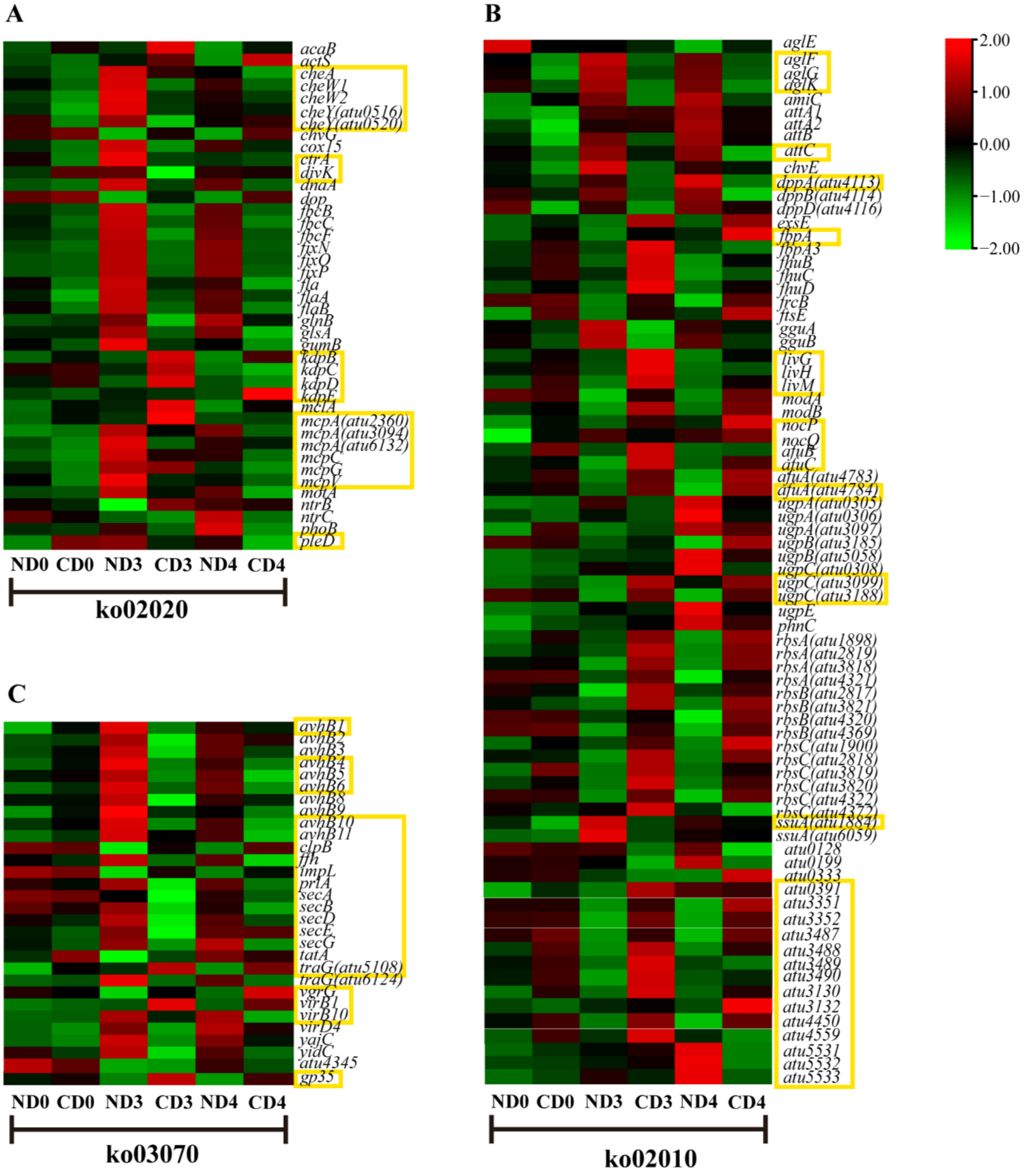

Figure 3. The expression of DEGs (in *Agrobacterium* cells treated with tea leaves) enriched in KEGG pathways (ko02020, ko02010, ko03070) assigned to the category environmental information processing. (**A**) ko02020, two-component system. (**B**) ko02010, ABC transporters. (**C**) ko03070, bacterial secretion system. The highlighted genes were discussed in more detail. The expression values were present as lg (FPKM). The highlighted genes are discussed in more detail.

The *tatA* (*atu1706*) involved in twin-arginine targeting (tat), *avhB1* (*atu5162*), and *traG* (*atu5108*) involved in T4SS were up-regulated by 1.41-fold, 1.83-fold, and 2.08-fold, respectively, when comparing tea to tobacco on day 0 (Figure 3C and Table S5). On day 3, the genes in the protein secretion system sec/SRP (*secA*, *secB*, *secD*, *secE*, *secG*) were all down-regulated in the tea group; T4SS genes were all down-regulated, except for *virB1* (*atu6171*) and *traG* (*atu5108*); T6SS genes, *impL* and *clpB*, were up-regulated (Figure 3C and Table S5). On day 4, the sec/SRP genes *secG*, *prlA* and *ffh* were down-regulated; T4SS

genes, *virB1* and *traG* were up-regulated but other T4SS genes (*avhB4*, *avhB5*, *avhB6*, *avhB10*, *avhB11*, *virB10*) were down-regulated; T6SS genes, *vgrG*, *impL* and *clpB* were up-regulated (Figure 3C). *Gp35* (*atu0956*) was up-regulated in the tea groups compared to the tobacco groups on days 3 and 4, by more than 77-fold and 47-fold, respectively (Table S5).

3.5.2. Expression Pattern Analysis of Genes Related to Cellular Processes

Amongst three enriched pathways (QS, flagellar assembly, and bacterial chemotaxis) under cellular processes, the QS pathway (ko02024) had the most enriched DEGs. In the QS pathway, the QQ regulator gene *traM* was induced immediately after co-cultivation, but was strongly suppressed on days 3 and 4 (Figure 4A). *traI*, involved in the synthesis of OC8-HSL [28], and *trb* genes (*atu6031–6035*, *atu6037–6041*) had a similar expression pattern to *traM* (Figure 4A). The gene *blcC* was up-regulated on days 3 and 4 (Figure 4A). Nine genes encoding GABA transporters (*atu1410–1413*, *atu3089*, *atu4123*, *atu4569*, *livM*, *amic*) were up-regulated in *Agrobacterium* on day 0; fourteen genes were up-regulated on day 3; six genes (*atu1838*, *atu1125*, *atu4123*, *atu4125*, *atu4126*, *atu4127*) were up-regulated on day 4 in the tea group, compared to the tobacco group (Figure 4A, Tables S4 and S5). A series of genes (*atu2514–2518*, *atu3433–3436*, *atu4620–4623*, and others) encoding the peptide/nickel transport system permease protein that promotes biofilm production [39] were differentially expressed (Figure 4A, Tables S4 and S5). On day 0 in the tea group, five genes were up-regulated while four were down-regulated; on day 3, thirty-nine genes were up-regulated and ten were down-regulated; on day 4, nineteen genes were up-regulated and eleven were down-regulated (Figure 4A, Table S5). Most of the genes (*phoB* = *atu0425*, *chvB* = *atu2730*, *exoY* = *atu3327*, *dcgA* = *atu1257*, *ros* = *atu0916*, *exoC* = *atu4074*, *exoB* = *atu4166*, *dcgB* = *atu1691*, *exoW* = *atu4058*, *crdR* = *atu0361*, *rrpX* = *atu1631*, *speF* = *atu3196*) related to EPS [40] showed a consistent down-regulation trend, while others (*pssA* = *atu0102*, *dcpA* = *atu3495*, *ppx1* = *atu0619*, *celB* = *atu3308*) were strongly up-regulated on day 3, in the tea group compared to the tobacco group (Table S5).

In the bacterial chemotaxis pathway (ko02030), *mcp* genes (*mcpV*, *mcpG*, *atu5442*), *cheA*, *cheW*, *cheY*, *atu3063*, *atu3533* and *dppA* were down-regulated in the tea group on day 0 (Figure 4B and Table S5). On day 3, six *mcp* genes (*mcpA*, *mcpC*, *mcpV*, *atu0373*, *atu5442*), *cheA*, *cheW*, *cheY*, *fliM*, *motA* and *dppA* were all down-regulated (Figure 4B and Table S5). On day 4, except for *mclA*, *motB* and *rbsB* genes, all the other genes enriched in this pathway were down-regulated (Figure 4B).

In the flagellar assembly pathway (ko02040), the *fla*, *flaA*, *flaB*, *flgD*, *flgK*, *flgL genes* were down-regulated, while *fliL* was up-regulated on day 0 (Figure 4C and Table S5). On day 3, all 23 *Agrobacterium* DEGs enriched in the pathway were down-regulated in the tea compared to the tobacco group (Table S5). On day 4, *motB* and *fliR* were up-regulated, and the other 21 DEGs enriched in the pathway were down-regulated in the tea compared to the tobacco group (Figure 4C, Tables S4 and S5).

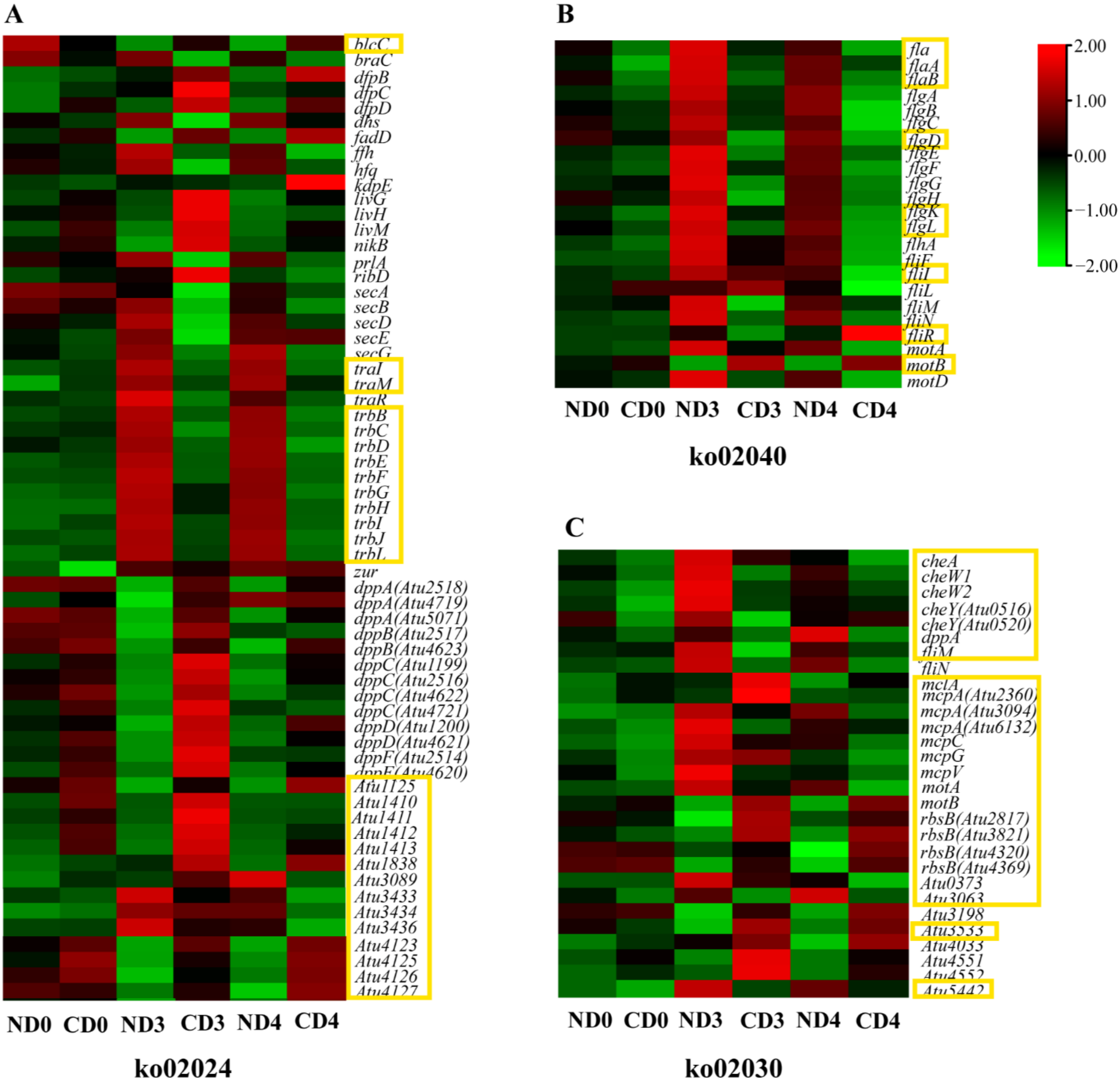

Figure 4. The expression of DEGs (in *Agrobacterium* cells co-cultured with tea leaves) enriched in KEGG pathways (ko02040, ko02024, ko02030) assigned to the category cellular processes. (**A**) ko02024, quorum sensing pathway. (**B**) ko02040, flagellar assembly pathway. (**C**) ko02030, bacterial chemotaxis pathway. The expression values were presented as lg (FPKM). The highlighted genes are discussed in detail.

3.6. Quantitative Reverse Transcription-PCR (qRT-PCR) Verification

To validate the data from transcriptomes, nine candidate genes and three housekeeping genes were selected to perform qRT-PCR. The comparison of transcriptome data (Figure 5A) and qRT-PCR data showed that *flaB*, *flaA*, and *dppA* were down-regulated in the tea groups from day 0 to day 4 (Figure 5B). On the other hand, the expressions of *virE0*, *upp*, *mcpV*, *cheW1*, and *cheW2* were up-regulated on day 0 in the tea group, compared to the tobacco group. The correlation analysis displayed a significant correlation ($p < 0.01$) between the RNA-seq and qRT-PCR data with a Pearson correlation coefficient of 0.912, which implied that the RNA-seq data were highly reliable (Figure 5C).

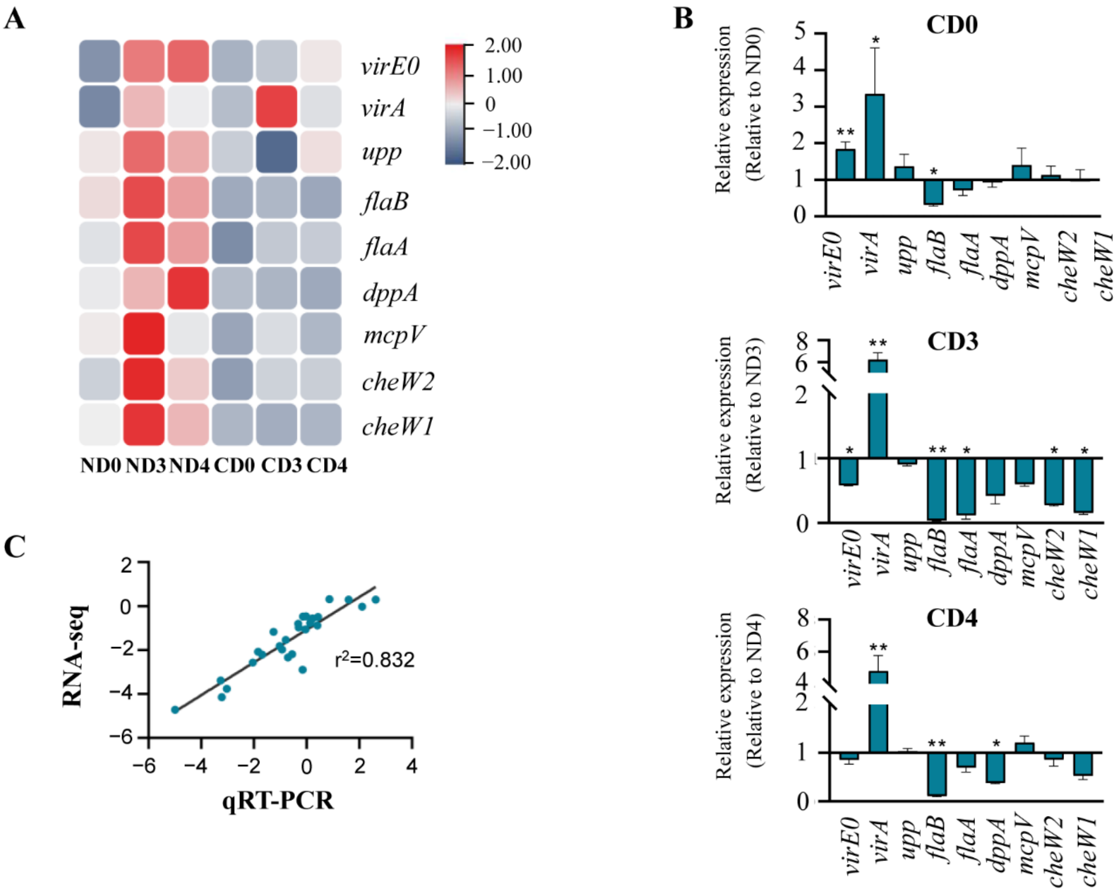

Figure 5. Validation of RNA-seq data using qRT-PCR. (**A**) Heatmap analysis. The data are based on the transcriptome results, and the expression values were presented as lg (FPKM). (**B**) Verification with qRT-PCR. The relative expression level was present as $2^{-\Delta\Delta Ct}$. * means $p \leq 0.05$ and ** means $p \leq 0.01$; the comparisons are between tea-leaf treatments (CD0, CD3, CD4) and corresponding tobacco leaf treatments (ND0, ND3, ND4). (**C**) Correlation analysis of the qRT-PCR and RNA-seq results. Pearson correlation coefficients = 0.912, $p < 0.01$.

4. Discussion

The efficiency of AMT is influenced by both biotic and abiotic factors. Abiotic factors include all the conditions from the pre-culture methods of the bacterium to transgenic plant screening. Biotic factors include the bacterium strain and the explant type. A slight alteration to these conditions can change biological activities in both *Agrobacterium* and in plant cells, affecting the genetic transformation efficiency through the pathogen–plant interaction. In this study, we focused on the pathogen–plant attachment, as well as the different transcriptional responses of the *Agrobacterium* to plants. Our results illustrate the general biological regulation mechanisms in *Agrobacterium* during the infection of explants. We found several possible pathways by which the efficiency of genetic transformation of tea plants was decreased compared to tobacco (Figure 6), based on SEM and transcriptome.

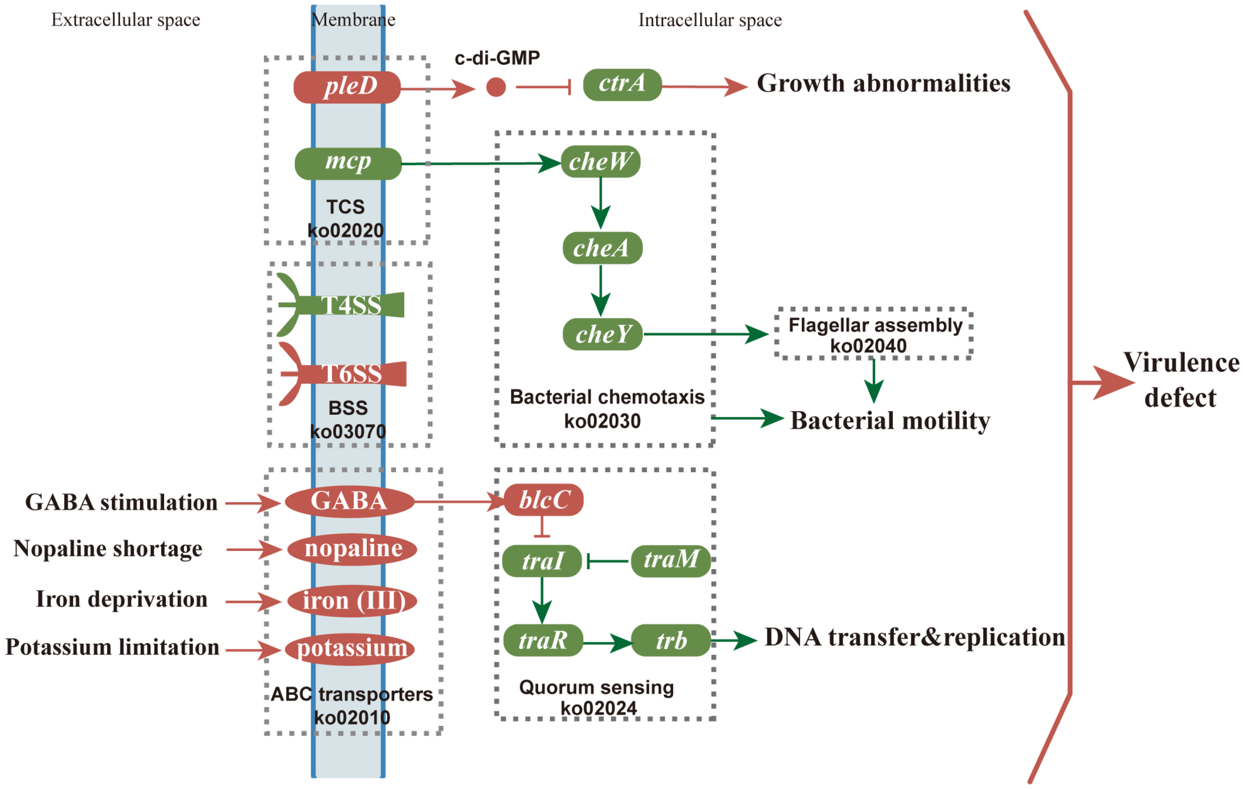

Figure 6. The brief regulatory model of Kyoto Encyclopedia of Genes and Genome (KEGG) pathways of *Agrobacterium* GV3101 in tea groups. The data were based on the transcriptome results. The round rectangle and ellipse nodes represent genes and transport systems, respectively. The boxes with gray dashed lines represent pathways. Pointed arrows indicate activation, and blunt arrows indicate repression. Red represents up-regulation, and green represents down-regulation.

In the tea group, we noticed various growth defects in *Agrobacterium*, such as ectopic growth poles, minicells, and branched cells, which could have been caused by an error in the cell-division machinery [41]. As a result, we observed several short and swollen minicells and big branched cells of *Agrobacterium* with several constriction sites (Figure 2B,D,E). These growth defects mentioned above suggest that the normal cell division of *Agrobacterium* was disrupted (directly or indirectly) by compounds secreted from tea leaves. *PleD* indirectly regulates *ctrA* expression to interfere with DNA replication, cell division, and morphogenesis [42–44]. This study found that the *pleD* in *Agrobacterium* from the tea-leaf group was up-regulated on the first day of co-cultivation, while *ctrA* was down-regulated (Figure 3A). Meanwhile, the genes related to genetic information processing were generally down-regulated (Figure S4). These results suggested that tea-leaf compounds influenced the expression of bacterial genetic activities and led to growth abnormalities in *Agrobacterium* cells (Figure 6). Since the genetic information processing was affected by compounds in the tea leaf, the transfer of exogenous genes might be inhibited.

In the transcriptome data, a series of genes contributing to both reversible and irreversible attachment were immediately down-regulated in the tea-leaf group (Table S4). The genes related to flagellar assembly, chemotaxis, UPP, and EPS production were inhibited in the tea groups (Figures 3A and 4C), which implied a failure of the bacteria to form a biofilm. It has been known since 1984 that "Nigerian chewing sticks" [45], rich in gallotannins, prevent the formation of bacterial film that causes plaque in teeth [45]. In the SEM images, the *Agrobacterium* cells were wrapped in cellulose fiber (Figure 2H) caused by an overproduction of cellulose, which agrees with our transcriptome data that showed an up-regulation (on day 4) of *celB* (Table S5), an essential gene for cellulose synthesis [46].

It has been established that overproduction of cellulose does not affect the virulence of *Agrobacterium*, but underproduction of UPP can lead to a fragile attachment [47].

Iron is an essential element for bacterium cell proliferation during a host infection [48], and its deficiency inhibits biofilm formation by *Agrobacterium* [49]. Ferritin enzymes from *A. tumefaciens* play a key role in bacterial full virulence by regulating iron homeostasis and oxidative stress survival [50]. Ferritins are enzymes that store iron as their core molecule, and iron deficiency and/or ferritin deficiency impair AMT. *Agrobacterium* has the following two ferritin-encoding genes: *atu2771* and *atu2477*, of which *atu2771* is annotated as a Bfr-encoding gene (Bacteriotransferrin, *bfr*) and *atu2477* is a Dps-encoding gene (DNA-binding protein from starved cells, Dps). Both *atu2771* and *atu2477* are reported to be responsible for iron homeostasis, oxidative stress resistance, and the growth of *A. tumefaciens* [50]. According to Renzett and his colleges [51], tea catechins are capable of iron chelation in *Escherichia coli* and *Pseudomonas fluorescens*. We found that in the tea groups, *bfr* (*atu2771*), *dps* (*atu2477*), and the ferric uptake regulator *fur* were strongly down-regulated (Table S5), and the genes (*afuA*, *afuB*, *afuC*) encoding iron (III) transport system were up-regulated, similar to the expression patterns of those genes in *Agrobacterium* under iron limitation [49]. Thus, we speculate that catechins produced in tea leaves might suppress *A. tumefaciens* growth (Figure 1) through a severe iron limitation triggered by interference with iron acquisition, storage, and chelation, all leading to the lack of bacterial iron homeostasis.

The TCS KdpD/KdpE are known to be involved in K^+ transport, which directly regulates bacterial virulence [52]. In our investigation, up-regulations of the *kdp* genes in the tea groups (Figure 3A) probably resulted from K^+ limitation. *Agrobacterium* could use nopaline, a plant-derived amino acid derivative, as a nutritional source in case of starvation stress [53,54]. The up-regulation of the nopaline transport system was found in the tea-leaf groups (Figure 3B), suggesting that the *Agrobacterium* cells in the tea groups were under low nutritional status. Moreover, other strongly up-regulated transport systems were also observed in the tea-leaf group, such as monosaccharides, polyols, and lipid transporters (Figure 3B, Tables S4 and S5). Therefore, our results suggested that *Agrobacterium* cells co-cultivated with tea leaves faced multiple major nutritional shortages, including iron restriction, potassium limitation, nitrogen (nopaline) deficiency, and other mineral deficiencies, as mentioned above. These deficiencies, isolated or combined, led to a significant reduction in bacterial virulence. All the DEGs enriched in oxidative phosphorylation, citrate (TCA) cycle, and ribosome pathways were inhibited in the tea-leaf groups compared to the tobacco groups (Table S4), which may indicate that the *Agrobacterium* cells lacked the energy required forseveral biological processes, especially for protein synthesis, based on the fact that they were all missing structural constituents of the ribosome (Figure S3). Furthermore, the up-regulation of the *recQ* gene, belonging to the SOS regulon [55], implied that *Agrobacteria* may have suffered severe DNA damage [56] in the presence of tea-leaf discs, which was consistent with a previous report [57] that epigallocatechin gallate (EGCG), one of the main catechin in tea leaves, caused iron limitation and SOS response in *Pseudomonas fluorescens*.

A successful AMT requires plant signals to activate VirA through phosphorylation. Subsequently, the phosphorylated VirA activates VirG, which regulates the transcription of downstream *vir* genes to form T4SS, the bacterial export system for T-DNA. In this study, *virD4* and *avhB* (homologous to *virB* genes) were down-regulated in *Agrobacterium* in the tea groups (Figure 3), which suggested that the essential step to AMT, T-DNA transfer, was blocked in tea leaves. Catechins, with their anti-microbial ability, were widely considered as a major restriction factor for the highly efficient AMT of tea plants [5,9,58,59]. Our SEM (Figure 1) and transcriptomic analysis (Figure 3) indicated that the suppression of the *Agrobacterium* growth and *vir* gene expression were observed in tea leaves, which were consistent with the report by Song et al. [58], who found that catechins severely reduced the *Agrobacterium* amount and *vir* gene transcripts. In order to improve the efficiency of tea plant AMT, the somatic embryos [5] and cotyledon callus [59], both with lower concentrations of catechins were used as explants. Although a transformation rate of 3.6%

was obtained [59], it did not meet the requirement of research and production of the tea industry. It also suggests that tea catechins are not the unique factors affecting the AMT of the tea plant, which leaves room to investigate other compounds produced by tea leaves that may be affecting the efficiency of AMT.

Intriguingly, the genes involved in T6SS, which have an intraspecific killing ability [21], were increased in the tea groups on days 3 and 4 (Figure 3). Another unusual gene regulation that drew our attention was the up-regulation of *gp35*. It has been reported that the Gp35 protein contains a putative cell wall hydrolase [60] that can cause *Agrobacterium* cell lysis. Thus, it is reasonable to speculate that the propagation of *Agrobacterium* was inhibited in the tea groups at a later stage of co-cultivation through the up-regulation of *gp35* and T6SS, which was consistent with the SEM data (Figure 2) in the present study.

Usually, a pathogen attack leads to the release of various antibacterial compounds by the host [61]. After sensing the antibacterial compounds, *Agrobacterium* reacts quickly by triggering a QQ regulator, *traM*, to stop TraR from binding to OC8-HSL, subsequently preventing transcription of conjugation-related genes [28]. In brief, plant-derived GABA up-regulates *blcC*, which encodes lactonase involved in the cleavage of OC8-HSL, strengthening the QQ process [28]. In the present study, the expression of GABA transporters was increased from day 0 to 4, and *blcC* was up-regulated on day 3, compared to the control (Table S5). Furthermore, the GABA concentration in wounded tea leaves is reported to be around 2.6 μg/g (fresh weight) and could be hundreds of times higher under anoxic conditions [62]. Therefore, we believe that the up-regulation of *blcC* in the tea group (Figure 4A) could be due to the increased level of GABA in the wounded tea leaves (leaf discs). The repression of the *trb* operon (Figure 4A) further supported our assumption that *Agrobacterium* relies on QQ to remove the previously induced QS signals induced by metabolism from tea leaves and to inhibit population growth, as well as plasmid transfer (Figure 6). The QQ function also explained why the bacterial population observed in the tea groups stayed constant over time in the control tobacco leaves (Figure 2).

5. Conclusions

Researchers have tried different methods to improve the efficiency of tea AMT, such as removing the tea polyphenols with polyvinylpyrrolidone (PVP) [8], adding L-glutamine [8] or L-glutamic acid [63]. However, no satisfactory result has been obtained so far, leaving researchers still searching for a successful AMT method for tea plants. This work provides the transcriptome landscape of *Agrobacterium* when co-cultured with tea leaves (*C. sinensis* (L.) O. Kuntze). Combining transcriptional results with SEM, we propose a model for *Agrobacterium* regulation that explains the low AMT efficiency in tea plants (Figure 6). Tea leaves release multiple antibacterial chemicals when wounded (e.g., catechins and GABA), which create an adverse environment for *Agrobacterium* through different inhibition mechanisms. The necessary biological processes for AMT, from energy acquisition to cell division, are disrupted so that bacterial cells develop a series of growth defects. These adverse shortcomings force *Agrobacterium* to minimize its population through QQ system activation (Figures 6 and 7). A large amount of GABA released by tea leaves also promotes QQ activity. Naturally, the bacterium virulence is weakened, or even eliminated, because of RNA translation failures and virulence protein shortages. In this paper, we raise an assumption that catechins and GABA in tea leaves were the most important factors that led to an unsuccessful tea AMT, through the inhibition of the plant–pathogen attachment, iron and potassium limitation, QS interruption, i.e., QQ system enhancement. Based on each pertinent mechanism being limited by catechins and GABA, we suggest that possible ways to improve tea AMT may include the selection of anti-GABA/catechins *Agrobacterium* strains, the reduction in GABA/catechins concentration in the medium, genetic control of *Agrobacterium* to enhance the QS process, and the addition of essential mineral elements, such as Fe and K, to help *Agrobacterium* maintain iron and potassium homeostasis in the presence of tea leaves. Nonaka et al. [64] increased AMT efficiency in tomatoes using

a new *A. tumefaciens* strain with GABA transaminase activity, which counteracted the GABA-triggered QQ process and enhanced QS process.

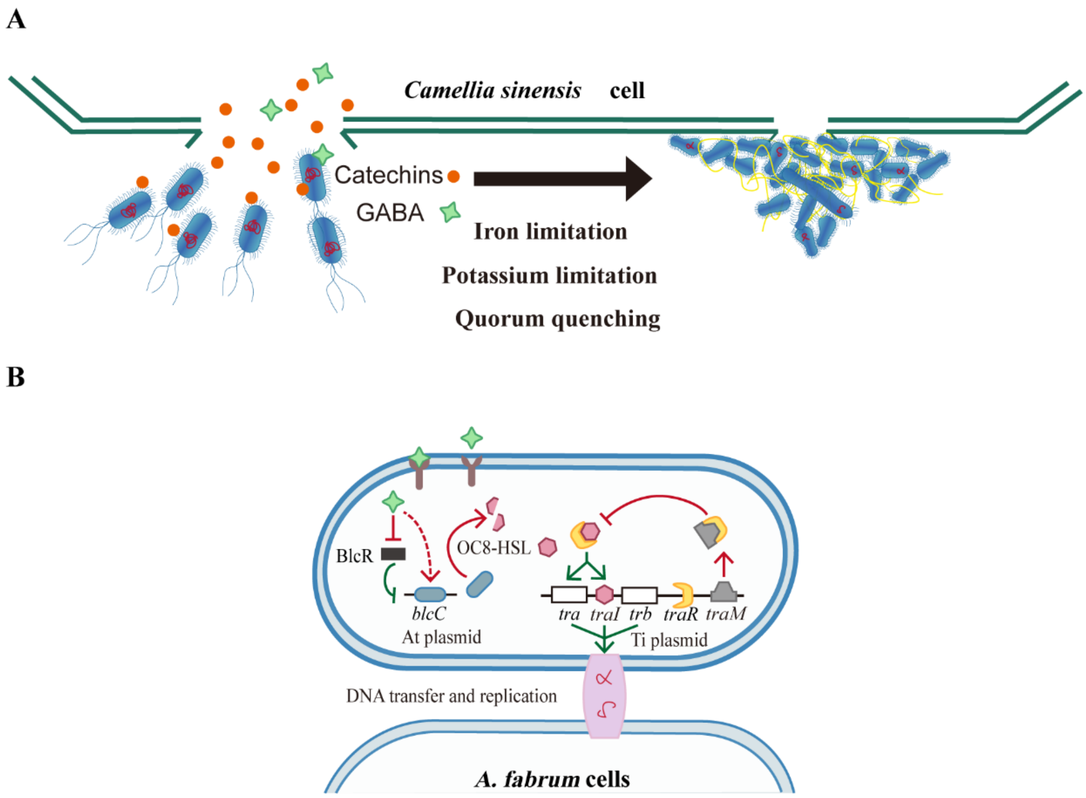

Figure 7. The regulation model of *Agrobacterium* response to tea leaves. (**A**) *Agrobacterium* cells are attracted by plant signals towards tea (*Camellia sinensis*) leaf cells. Tea-derived compounds, mainly catechins and gamma-aminobutyrate (GABA), induce iron limitation, potassium limitation, and quorum quenching (QQ) in *Agrobacteria*, which result in fragile plant–pathogen attachments, bacterial growth defects (branched cells and minicells with inaccurate genetic information), finally hindering AMT efficiency. (**B**) QQ process triggered by tea-derived GABA. GABA is imported into bacterial cells by Bra/atu2422 and inhibits transcriptional repressor BlcR; BlcR represses *blcC* gene; *blcC* encodes the lactonase, which cleaves OC8-HSL. Hence, GABA promotes *blcC* gene expression and OC8-HSL degradation. OC8-HSL binds to TraR, and the TraR-OC8-HSL complex activates the expression of *tra*, *trb* operon, and *traI*, all of which encode DNA transfer and replication system. TraM can also bind to TraR and compete with OC8-HSL. TraM expression and GABA import enhance the QQ system. As a result, the new bacterial cells might be injected with inaccurate (or no) genetic information. Pointed arrows indicate activation, and blunt arrows indicate repression. Red represents up-regulation, and the green represents down-regulation.

Although the changes in morphology and transcriptome profile in *Agrobacterium* were analyzed in the present work, additional work is warranted to obtain a complete picture of the mechanisms of recalcitrance in the AMT of tea plants. For example, different tea genotypes and *Agrobacterium* strains could be analyzed.

Supplementary Materials: The following supporting information can be downloaded at: https://www.mdpi.com/article/10.3390/biom12050688/s1, Figure S1: Scanning electron microscopic observation of *Agrobacterium* GV3101 attached to the cross-sections of tobacco and tea leaves., Figure S2: Quantity and located replicons of differentially expressed genes (DEGs) in different comparisons, Figure S3: Gene Ontology (GO) enrichment bubble charts of three comparisons: CD0 (*Agrobacterium* cells co-cultivated with *Camellia sinensis* (leaves) for 30 min) vs. ND0 (*Agrobacterium* cells co-cultivated with *Nicotiana benthamiana* (leaves) for 30 min); CD3 (*Agrobacterium* cells co-cultivated with *C. sinensis* for 3 d) vs. ND3 (*Agrobacterium* cells co-cultivated with *N. benthamiana* for 3 d); CD4 (*Agrobacterium* cells co-cultivated with *C. sinensis* for 4 d) vs. ND4 (*Agrobacterium* cells co-cultivated with *N. benthamiana* for 4 d), Figure S4: Kyoto Encyclopedia of Genes and Genome (KEGG) enrichment circle plots for the top 30 enriched pathways of three comparisons: CD0 (*Agrobacterium* cells co-cultivated with phylloplane of *Camellia sinensis* for 30 min) vs. ND0 (*Agrobacterium* cells co-cultivated with phylloplane of *N. benthamiana* for 30 min); CD3 (*Agrobacterium* cells co-cultivated with phylloplane of *C. sinensis* for 3 d) vs. ND3 (*Agrobacterium* cells co-cultivated with phylloplane of *N. benthamiana* for 3 d); CD4 (*Agrobacterium* cells co-cultivated with phylloplane of *C. sinensis* for 4 d) vs. ND4 (*Agrobacterium* cells co-cultivated with phylloplane of *N. benthamiana* for 4 d), Table S1: Primers used in the qRT-PCR reaction, Table S2: RNA-seq results of mapping rate of raw reads against the reference genome of *Agrobacterium tumefaciens* str. C58 plus and the plasmid pMKV060, Table S3: Results of Gene ontology (GO) enrichment analysis., Table S4: Results of Kyoto Encyclopedia of Genes and Genome (KEGG) enrichment analysis., Table S5: Fragments per kilobase of transcript sequence per million base pairs sequenced (FPKM) value of all genes in *Agrobacterium* GV3101.

Author Contributions: Conceptualization, S.L., J.H. and Z.L.; methodology, K.J. and N.T.; validation, K.J. and N.T.; formal analysis, K.J. and N.T.; investigation, L.X., M.G., Y.L., X.Z. and G.L.; writing—original draft preparation, K.J. and N.T.; writing—review, J.F.d.S.F. and D.S.; visualization, K.J., N.T., J.F.d.S.F. and D.S.; supervision, S.L., J.H. and Z.L.; project administration, S.L., J.H. and Z.L.; funding acquisition, S.L., J.H. and Z.L. All authors have read and agreed to the published version of the manuscript.

Funding: This research was funded by the National Natural Science Foundation of China (32172629, U19A2030, 31670689), National Key Research and Development Program of China (No. 2021YFD1200200), Provincial Natural Science Foundation of Hunan (2020JJ4358) and Hunan Provincial Seed Industry Innovation Project (2021NK1008).

Institutional Review Board Statement: Not applicable.

Informed Consent Statement: Not applicable.

Data Availability Statement: The data presented in the study are included in the article/Supplementary Material; further inquiries can be directed to the corresponding author.

Conflicts of Interest: The authors declare no conflict of interest. The funders had no role in the design of the study; in the collection, analyses, or interpretation of data; in the writing of the manuscript, or in the decision to publish the results.

References

1. Prasanth, M.I.; Sivamaruthi, B.S.; Chaiyasut, C.; Tencomnao, T. A review of the role of green tea (*Camellia sinensis*) in antiphotoaging, stress resistance, neuroprotection, and autophagy. *Nutrients* **2019**, *11*, 474. [CrossRef] [PubMed]
2. Mondal, T.K.; Bhattacharya, A.; Laxmikumaran, M.; Singh Ahuja, P. Recent advances of tea (*Camellia sinensis*) biotechnology. *Plant Cell Tissue Organ Cult.* **2004**, *76*, 195–254. [CrossRef]
3. Butelli, E.; Titta, L.; Giorgio, M.; Mock, H.-P.; Matros, A.; Peterek, S.; Schijlen, E.G.W.M.; Hall, R.D.; Bovy, A.G.; Luo, J.; et al. Enrichment of tomato fruit with health-promoting anthocyanins by expression of select transcription factors. *Nat. Biotechnol.* **2008**, *26*, 1301–1308. [CrossRef] [PubMed]
4. Chilcoat, D.; Liu, Z.-B.; Sander, J. Chapter Two-Use of CRISPR/Cas9 for Crop Improvement in Maize and Soybean. In *Progress in Molecular Biology and Translational Science*; Weeks, D.P., Yang, B., Eds.; Academic Press: Cambridge, MA, USA, 2017; Volume 149, pp. 27–46. [CrossRef]
5. Mondal, T.; Bhattacharya, A.; Ahuja, P.; Chand, P. Transgenic tea [*Camellia sinensis* (L.) O. Kuntze cv. Kangra Jat] plants obtained by *Agrobacterium*-mediated transformation of somatic embryos. *Plant Cell Rep.* **2001**, *20*, 712–720. [CrossRef]
6. Singh, H.R.; Bhattacharyya, N.; Agarwala, N.; Bhagawati, P.; Deka, M.; Das, S. Exogenous gene transfer in Assam tea [*Camellia assamica* (Masters)] by *Agrobacterium*-mediated transformation using somatic embryo. *Eur. J. Exp. Biol.* **2014**, *4*, 166–175.

7. Singh, H.R.; Hazarika, P.; Agarwala, N.; Bhattacharyya, N.; Bhagawati, P.; Gohain, B.; Bandyopadhyay, T.; Bharalee, R.; Gupta, S.; Deka, M.; et al. Transgenic tea over-expressing *Solanum tuberosum* endo-1,3-beta-d-glucanase gene conferred resistance against blister blight disease. *Plant Mol. Biol. Rep.* **2018**, *36*, 107–122. [CrossRef]
8. Sandal, I.; Saini, U.; Lacroix, B.; Bhattacharya, A.; Ahuja, P.S.; Citovsky, V. *Agrobacterium*-mediated genetic transformation of tea leaf explants: Effects of counteracting bactericidity of leaf polyphenols without loss of bacterial virulence. *Plant Cell Rep.* **2007**, *26*, 169–176. [CrossRef]
9. Xia, E.-H.; Zhang, H.-B.; Sheng, J.; Li, K.; Zhang, Q.-J.; Kim, C.; Zhang, Y.; Liu, Y.; Zhu, T.; Li, W.; et al. The tea tree genome provides insights into tea flavor and independent evolution of caffeine biosynthesis. *Mol. Plant* **2017**, *10*, 866–877. [CrossRef]
10. Mukhopadhyay, M.; Mondal, T.; Chand, P. Biotechnological advances in tea (*Camellia sinensis* [L.] O. Kuntze): A review. *Plant Cell Rep.* **2015**, *35*, 255–287. [CrossRef]
11. Mohanpuria, P.; Kumar, V.; Ahuja, P.S.; Yadav, S.K. *Agrobacterium*-mediated silencing of caffeine synthesis through root transformation in *Camellia sinensis* L. *Mol. Biotechnol.* **2011**, *48*, 235–243. [CrossRef]
12. Matsumoto, S.; Fukui, M. Effect of acetosyringone application on *Agrobacterium*-mediated gene transfer in tea plant (*Camellia sinensis*). *Bull. Natl. Res. Inst. Veg. Ornam. Plants Tea Jpn.* **1999**, *14*, 9–15.
13. Lopez, S.J.; Kumar, R.R.; Pius, P.K.; Muraleedharan, N. *Agrobacterium tumefaciens*-Mediated genetic transformation in tea (*Camellia sinensis* [L.] O. Kuntze). *Plant Mol. Biol. Rep.* **2004**, *22*, 201–202. [CrossRef]
14. Gelvin, S.B. *Agrobacterium*-mediated plant transformation: The biology behind the "gene-jockeying" tool. *Microbiol. Mol. Biol. Rev.* **2003**, *67*, 16–37. [CrossRef] [PubMed]
15. Thompson, M.G.; Moore, W.M.; Hummel, N.F.C.; Pearson, A.N.; Barnum, C.R.; Scheller, H.V.; Shih, P.M. *Agrobacterium tumefaciens*: A bacterium primed for synthetic biology. *BioDes. Res.* **2020**, *2020*, 8189219. [CrossRef]
16. Guo, M.; Huang, Z.; Yang, J. Is there any crosstalk between the chemotaxis and virulence induction signaling in *Agrobacterium tumefaciens*? *Biotechnol. Adv.* **2017**, *35*, 505–511. [CrossRef]
17. Matthysse, A.G. Attachment of *Agrobacterium* to plant surfaces. *Front. Plant Sci.* **2014**, *5*, 252. [CrossRef]
18. Dazzo, F.; Truchet, G.; Sherwood, J.; Hrabak, E.; Abe, M.; Pankratz, S. Specific phases of root hair attachment in the *Rhizobium trifolii*-clover symbiosis. *Appl. Environ. Microbiol.* **1984**, *48*, 1140–1150. [CrossRef]
19. Heindl, J.E.; Wang, Y.; Heckel, B.C.; Mohari, B.; Feirer, N.; Fuqua, C. Mechanisms and regulation of surface interactions and biofilm formation in *Agrobacterium*. *Front. Plant Sci.* **2014**, *5*, 176. [CrossRef]
20. Bernal, P.; Llamas, M.A.; Filloux, A. Type VI secretion systems in plant-associated bacteria. *Environ. Microbiol.* **2018**, *20*, 1–15. [CrossRef]
21. Yu, M.; Wang, Y.-C.; Huang, C.-J.; Ma, L.-S.; Lai, E.-M. *Agrobacterium tumefaciens* deploys a versatile antibacterial strategy to increase its competitiveness. *J. Bacteriol.* **2021**, *203*, e00490-20. [CrossRef]
22. Dong, T.G.; Dong, S.; Catalano, C.; Moore, R.; Liang, X.; Mekalanos, J.J. Generation of reactive oxygen species by lethal attacks from competing microbes. *Proc. Natl. Acad. Sci. USA* **2015**, *112*, 2181–2186. [CrossRef]
23. Silva, L.N.; Zimmer, K.R.; Macedo, A.J.; Trentin, D.S. Plant natural products targeting bacterial virulence factors. *Chem. Rev.* **2016**, *116*, 9162–9236. [CrossRef] [PubMed]
24. Mukherjee, S.; Bassler, B.L. Bacterial quorum sensing in complex and dynamically changing environments. *Nat. Rev. Microbiol.* **2019**, *17*, 371–382. [CrossRef] [PubMed]
25. Zhang, L.; Murphy, P.J.; Kerr, A.; Tate, M.E. *Agrobacterium* conjugation and gene regulation by N-acyl-L-homoserine lactones. *Nature* **1993**, *362*, 446–448. [CrossRef] [PubMed]
26. Grandclément, C.; Tannières, M.; Moréra, S.; Dessaux, Y.; Faure, D. Quorum quenching: Role in nature and applied developments. *FEMS Microbiol. Rev.* **2015**, *40*, 86–116. [CrossRef] [PubMed]
27. White, C.E.; Winans, S.C. Identification of amino acid residues of the *Agrobacterium tumefaciens* quorum-sensing regulator TraR that are critical for positive control of transcription. *Mol. Microbiol.* **2005**, *55*, 1473–1486. [CrossRef] [PubMed]
28. Dessaux, Y.; Faure, D. Quorum sensing and quorum quenching in *Agrobacterium*: A "Go/No Go System"? *Genes* **2018**, *9*, 210. [CrossRef] [PubMed]
29. Zhang, J.-W.; Xuan, C.-G.; Lu, C.-H.; Guo, S.; Yu, J.-F.; Asif, M.; Jiang, W.-J.; Zhou, Z.-G.; Luo, Z.-Q.; Zhang, L.-Q. AidB, a novel thermostable *N*-acylhomoserine lactonase from the bacterium *Bosea* sp. *Appl. Environ. Microbiol.* **2019**, *85*, e02065-19. [CrossRef]
30. Haudecoeur, E.; Faure, D. A fine control of quorum-sensing communication in *Agrobacterium tumefaciens*. *Commun. Integr. Biol.* **2010**, *3*, 84–88. [CrossRef]
31. Haudecoeur, E.; Planamente, S.; Cirou, A.; Tannières, M.; Shelp, B.J.; Moréra, S.; Faure, D. Proline antagonizes GABA-induced quenching of quorum-sensing in *Agrobacterium tumefaciens*. *Proc. Natl. Acad. Sci. USA* **2009**, *106*, 14587–14592. [CrossRef]
32. Planamente, S.; Vigouroux, A.; Mondy, S.; Aumont-Nicaise, M.; Faure, D.; Moréra, S. A conserved mechanism of GABA binding and antagonism is revealed by structure-function analysis of the periplasmic binding protein Atu2422 in *Agrobacterium tumefaciens*. *J. Biol. Chem.* **2010**, *285*, 30294–30303. [CrossRef] [PubMed]
33. Engström, P.; Zambryski, P.; Van Montagu, M.; Stachel, S. Characterization of *Agrobacterium tumefaciens* virulence proteins induced by the plant factor acetosyringone. *J. Mol. Biol.* **1987**, *197*, 635–645. [CrossRef]
34. Bourras, S.; Rouxel, T.; Meyer, M. *Agrobacterium tumefaciens* gene transfer: How a plant pathogen hacks the nuclei of plant and nonplant organisms. *Phytopathology* **2015**, *105*, 1288–1301. [CrossRef] [PubMed]

35. Pathi, K.M.; Tula, S.; Tuteja, N. High frequency regeneration via direct somatic embryogenesis and efficient *Agrobacterium*-mediated genetic transformation of tobacco. *Plant Signal. Behav.* **2013**, *8*, e24354. [CrossRef]
36. Maher, M.F.; Nasti, R.A.; Vollbrecht, M.; Starker, C.G.; Clark, M.D.; Voytas, D.F. Plant gene editing through de novo induction of meristems. *Nat. Biotechnol.* **2020**, *38*, 84–89. [CrossRef]
37. Benjamini, Y.; Hochberg, Y. Controlling the false discovery rate: A practical and powerful approach to multiple testing. *J. R. Stat. Soc. Ser. B Stat. Methodol.* **1995**, *57*, 289–300. [CrossRef]
38. Xie, C.; Mao, X.; Huang, J.; Ding, Y.; Wu, J.; Dong, S.; Kong, L.; Gao, G.; Li, C.-Y.; Wei, L. KOBAS 2.0: A web server for annotation and identification of enriched pathways and diseases. *Nucleic Acids Res.* **2011**, *39*, W316–W322. [CrossRef]
39. Wood, D.W.; Setubal, J.C.; Kaul, R.; Monks, D.E.; Kitajima, J.P.; Okura, V.K.; Zhou, Y.; Chen, L.; Wood, G.E.; Almeida, N.F.; et al. The genome of the natural genetic engineer *Agrobacterium tumefaciens* C58. *Science* **2001**, *294*, 2317–2323. [CrossRef]
40. Matthysse, A.G. Exopolysaccharides of *Agrobacterium tumefaciens*. In *Agrobacterium Biology: From Basic Science to Biotechnology*; Gelvin, S.B., Ed.; Springer International Publishing: Cham, Switzerland, 2018; pp. 111–141. [CrossRef]
41. Flores, S.A.; Howell, M.; Daniel, J.J.; Piccolo, R.; Brown, P.J.B. Absence of the Min system does not cause major cell division defects in *Agrobacterium tumefaciens*. *Front. Microbiol.* **2018**, *9*, 681. [CrossRef]
42. Kaczmarczyk, A.; Hempel, A.M.; von Arx, C.; Böhm, R.; Dubey, B.N.; Nesper, J.; Schirmer, T.; Hiller, S.; Jenal, U. Precise timing of transcription by c-di-GMP coordinates cell cycle and morphogenesis in *Caulobacter*. *Nat. Commun.* **2020**, *11*, 816. [CrossRef]
43. Paul, R.; Jaeger, T.; Abel, S.; Wiederkehr, I.; Folcher, M.; Biondi, E.G.; Laub, M.T.; Jenal, U. Allosteric regulation of histidine kinases by their cognate response regulator determines cell fate. *Cell* **2008**, *133*, 452–461. [CrossRef] [PubMed]
44. Farrera-Calderon, R.G.; Pallegar, P.; Westbye, A.B.; Wiesmann, C.; Lang, A.S.; Beatty, J.T. The CckA-ChpT-CtrA phosphorelay controlling *Rhodobacter capsulatus* gene transfer agent production is bidirectional and regulated by cyclic di-GMP. *J. Bacteriol.* **2021**, *203*, e00525-20. [CrossRef] [PubMed]
45. Wolinsky, L.E.; Sote, E.O. Isolation of natural plaque-inhibiting substances from 'Nigerian chewing sticks'. *Caries Res.* **1984**, *18*, 216–225. [CrossRef] [PubMed]
46. Matthysse, A.G.; White, S.; Lightfoot, R. Genes required for cellulose synthesis in *Agrobacterium tumefaciens*. *J. Bacteriol.* **1995**, *177*, 1069–1075. [CrossRef] [PubMed]
47. Barnhart, D.M.; Su, S.; Baccaro, B.E.; Banta, L.M.; Farrand, S.K. CelR, an ortholog of the diguanylate cyclase PleD of Caulobacter, regulates cellulose synthesis in *Agrobacterium tumefaciens*. *Appl. Environ. Microbiol.* **2013**, *79*, 7188–7202. [CrossRef]
48. Pandey, S.S.; Patnana, P.K.; Lomada, S.K.; Tomar, A.; Chatterjee, S. Co-regulation of iron metabolism and virulence associated functions by iron and XibR, a novel iron binding transcription factor, in the plant pathogen *Xanthomonas*. *PLoS Pathog.* **2016**, *12*, e1006019. [CrossRef]
49. Heindl, J.E.; Hibbing, M.E.; Xu, J.; Natarajan, R.; Buechlein, A.M.; Fuqua, C. Discrete responses to limitation for iron and manganese in *Agrobacterium tumefaciens*: Influence on attachment and biofilm formation. *J. Bacteriol.* **2015**, *198*, 816–829. [CrossRef]
50. Yang, J.; Pan, X.; Xu, Y.; Li, Y.; Xu, N.; Huang, Z.; Ye, J.; Gao, D.; Guo, M. *Agrobacterium tumefaciens* ferritins play an important role in full virulence through regulating iron homeostasis and oxidative stress survival. *Mol. Plant Pathol.* **2020**, *21*, 1167–1178. [CrossRef]
51. Renzetti, A.; Betts, J.W.; Fukumoto, K.; Rutherford, R.N. Antibacterial green tea catechins from a molecular perspective: Mechanisms of action and structure–activity relationships. *Food Funct.* **2020**, *11*, 9370–9396. [CrossRef]
52. Freeman, Z.N.; Dorus, S.; Waterfield, N.R. The KdpD/KdpE two-component system: Integrating K+ homeostasis and virulence. *PLoS Pathog.* **2013**, *9*, e1003201. [CrossRef]
53. Zanker, H.; von Lintig, J.; Schröder, J. Opine transport genes in the octopine (*occ*) and nopaline (*noc*) catabolic regions in Ti plasmids of *Agrobacterium tumefaciens*. *J. Bacteriol.* **1992**, *174*, 841–849. [CrossRef] [PubMed]
54. Hosie, A.H.F.; Poole, P.S. Bacterial ABC transporters of amino acids. *Res. Microbiol.* **2001**, *152*, 259–270. [CrossRef]
55. Irino, N.; Nakayama, K.; Nakayama, H. The *recQ* gene of *Escherichia coli* K12: Primary structure and evidence for SOS regulation. *Mol. Genet. Genom.* **1986**, *205*, 298–304. [CrossRef]
56. Baharoglu, Z.; Mazel, D. SOS, the formidable strategy of bacteria against aggressions. *FEMS Microbiol. Rev.* **2014**, *38*, 1126–1145. [CrossRef]
57. Liu, X.; Shen, B.; Du, P.; Wang, N.; Wang, J.; Li, J.; Sun, A. Transcriptomic analysis of the response of *Pseudomonas fluorescens* to epigallocatechin gallate by RNA-seq. *PLoS ONE* **2017**, *12*, e0177938. [CrossRef] [PubMed]
58. Song, D.; Feng, L.; Rana, M.; Gao, M.; Wei, S. Effects of catechins on *Agrobacterium*-mediated genetic transformation of *Camellia sinensis*. *Plant Cell Tissue Organ Cult.* **2014**, *119*, 27–37. [CrossRef]
59. Lv, Q.; Chen, C.; Xu, Y.; Hu, S.; Wang, L.; Sun, K.; Chen, X.; Li, X. Optimization of *Agrobacterium* tumefaciens-Mediated Transformation Systems in Tea Plant (*Camellia sinensis*). *Hortic. Plant J.* **2017**, *3*, 105–109. [CrossRef]
60. Attai, H.; Rimbey, J.; Smith, G.P.; Brown, P.J.B. Expression of a peptidoglycan hydrolase from lytic bacteriophages Atu_ph02 and Atu_ph03 triggers lysis of *Agrobacterium tumefaciens*. *Appl. Environ. Microbiol.* **2017**, *83*, e01498-17. [CrossRef]
61. Tannières, M.; Lang, J.; Barnier, C.; Shykoff, J.A.; Faure, D. Quorum-quenching limits quorum-sensing exploitation by signal-negative invaders. *Sci. Rep.* **2017**, *7*, 40126. [CrossRef]

62. Mei, X.; Chen, Y.; Zhang, L.; Fu, X.; Wei, Q.; Grierson, D.; Zhou, Y.; Huang, Y.; Dong, F.; Yang, Z. Dual mechanisms regulating glutamate decarboxylases and accumulation of gamma-aminobutyric acid in tea (*Camellia sinensis*) leaves exposed to multiple stresses. *Sci. Rep.* **2016**, *6*, 23685. [CrossRef]
63. Kumar, N.; Gulati, A.; Bhattacharya, A. L-glutamine and l-glutamic acid facilitate successful *Agrobacterium* Infection of recalcitrant tea cultivars. *Appl. Biochem. Biotechnol.* **2013**, *170*, 1649–1664. [CrossRef] [PubMed]
64. Nonaka, S.; Someya, T.; Zhou, S.; Takayama, M.; Nakamura, K.; Ezura, H. An *Agrobacterium tumefaciens* strain with gamma-aminobutyric acid transaminase activity shows an enhanced genetic transformation ability in plants. *Sci. Rep.* **2017**, *7*, 42649. [CrossRef] [PubMed]
