# Supplementary material for: Comparative Transcriptome Analysis of Agrobacterium tumefaciens Reveals the Molecular Basis for the Recalcitrant Genetic Transformation of Camellia sinensis L
Source: Biomolecules. 2022 May 11;12(5):688. doi: 10.3390/biom12050688 (PMC9138961; doi:10.3390/biom12050688)
Supplement: Supplementary file 1 [file biomolecules-12-00688-s001.zip › biomolecules-1645209-supplementary.zip!/Figure S1.pdf]

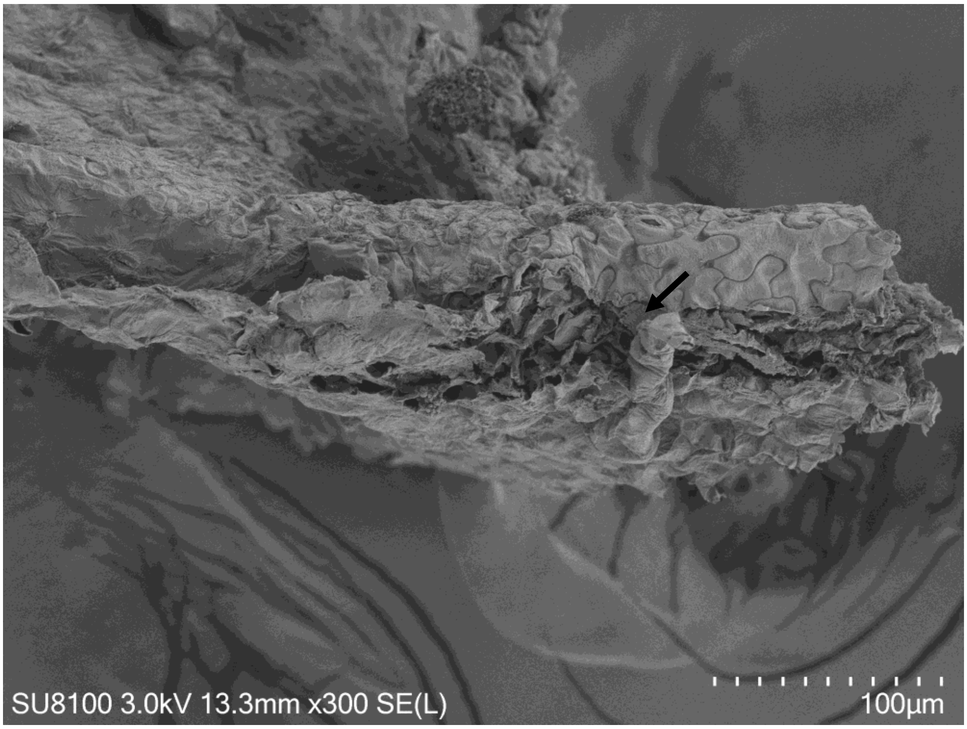

B

Agrobacterium on tea leaves

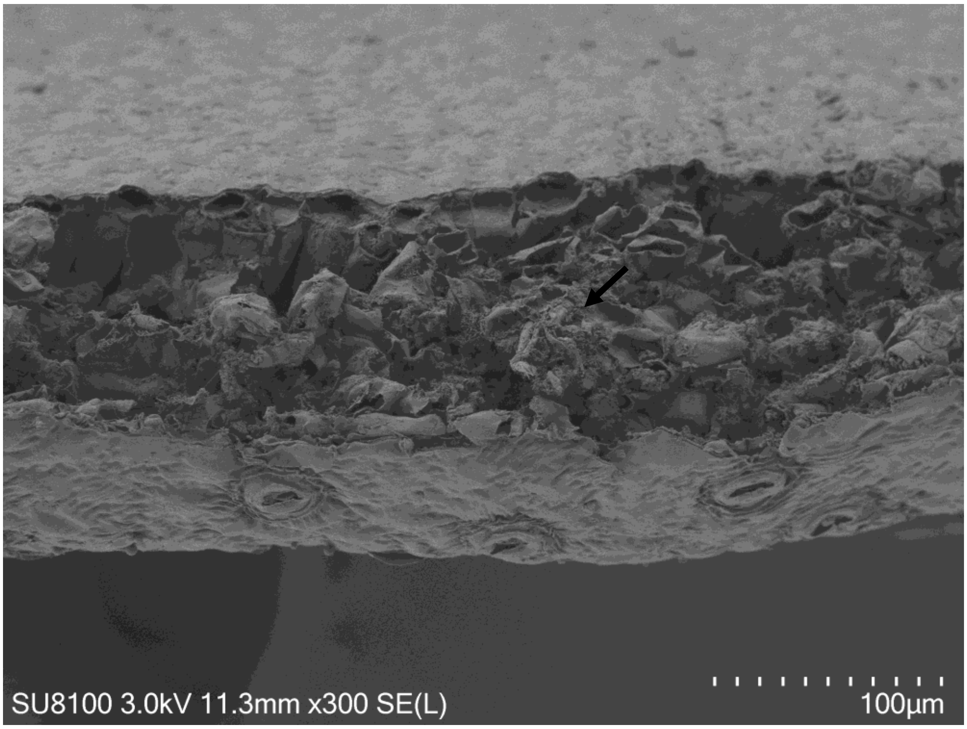

Figure S1 Scanning electron microscopic observation of *Agrobacterium* GV3101 attached to the cross-sections of tobacco and tea leaves. Compared with tobacco, there were more bacteria attached to the cross-section of tea leaves. Solid black arrowheads point to bacterium clusters.
