# Supplementary material for: Comparative Transcriptome Analysis of Agrobacterium tumefaciens Reveals the Molecular Basis for the Recalcitrant Genetic Transformation of Camellia sinensis L
Source: Biomolecules. 2022 May 11;12(5):688. doi: 10.3390/biom12050688 (PMC9138961; doi:10.3390/biom12050688)
Supplement: Supplementary file 1 [file biomolecules-12-00688-s001.zip › biomolecules-1645209-supplementary.zip!/Figure S2.pdf]

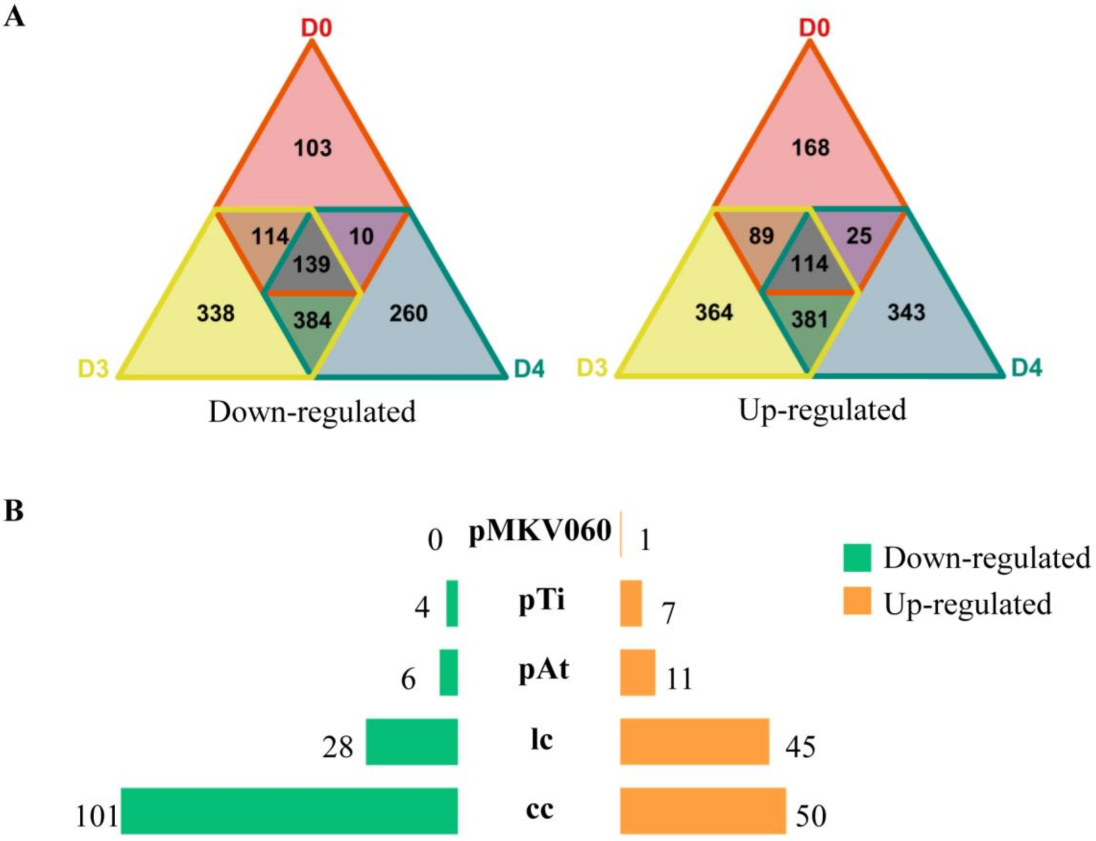


**Figure S2** Quantity and located replicons of differentially expressed genes (DEGs) in different comparisons. (A) Venn diagrams of *Agrobacterium* DEGs in *Agrobacterium*-tea groups compared to *Agrobacterium*-tobacco groups at different time points. (B) The located replicons of *Agrobacterium*-tea groups compared to *Agrobacterium*-tobacco groups at different time points. There are 5 replicons in *Agrobacterium* in this study: pMKV060: pMKV060 plasmid; Ti: tumor-inducing plasmid; At: At plasmid; lc: linear chromosome; cc: circular chromosome..
