# Supplementary material for: Comparative Transcriptome Analysis of Agrobacterium tumefaciens Reveals the Molecular Basis for the Recalcitrant Genetic Transformation of Camellia sinensis L
Source: Biomolecules. 2022 May 11;12(5):688. doi: 10.3390/biom12050688 (PMC9138961; doi:10.3390/biom12050688)
Supplement: Supplementary file 1 [file biomolecules-12-00688-s001.zip › biomolecules-1645209-supplementary.zip!/Figure S3.pdf]

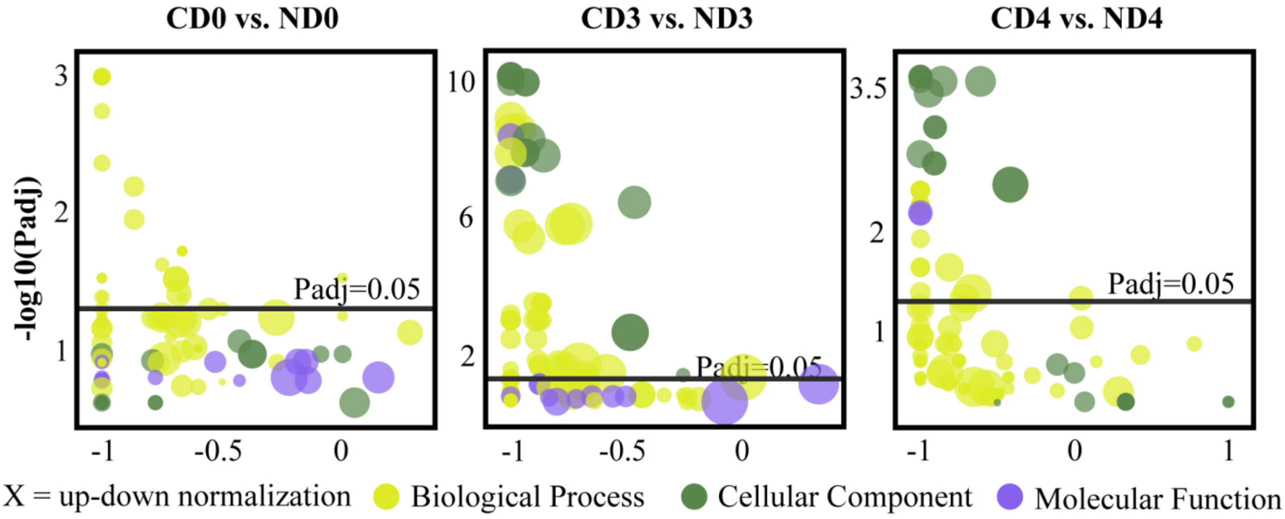

Figure S3 Gene Ontology (GO) enrichment bubble charts of three comparisons: CD0 (*Agrobacterium* cells co-cultivated with *Camellia sinensis* (leaves) for 30 min) vs. ND0 (*Agrobacterium* cells co-cultivated with *Nicotiana benthamiana* (leaves) for 30 min); CD3 (*Agrobacterium* cells co-cultivated with *C. sinensis* for 3 d) vs. ND3 (*Agrobacterium* cells co-cultivated with *N. benthamiana* for 3 d); CD4 (*Agrobacterium* cells co-cultivated with *C. sinensis* for 4 d) vs. ND4 (*Agrobacterium* cells co-cultivated with *N. benthamiana* for 4 d). Up-down normalization = (number of up-regulated genes - number of down-regulated genes)/(number of DEGs). Each bubble represents a GO term; different colors represent categories; the size of the bubble indicates the quantity of the genes annotated to the GO term; bubbles above the threshold line are considered significantly enriched GO terms.
