# Supplementary material for: Comparative Transcriptome Analysis of Agrobacterium tumefaciens Reveals the Molecular Basis for the Recalcitrant Genetic Transformation of Camellia sinensis L
Source: Biomolecules. 2022 May 11;12(5):688. doi: 10.3390/biom12050688 (PMC9138961; doi:10.3390/biom12050688)
Supplement: Supplementary file 1 [file biomolecules-12-00688-s001.zip › biomolecules-1645209-supplementary.zip!/Figure S4.pdf]

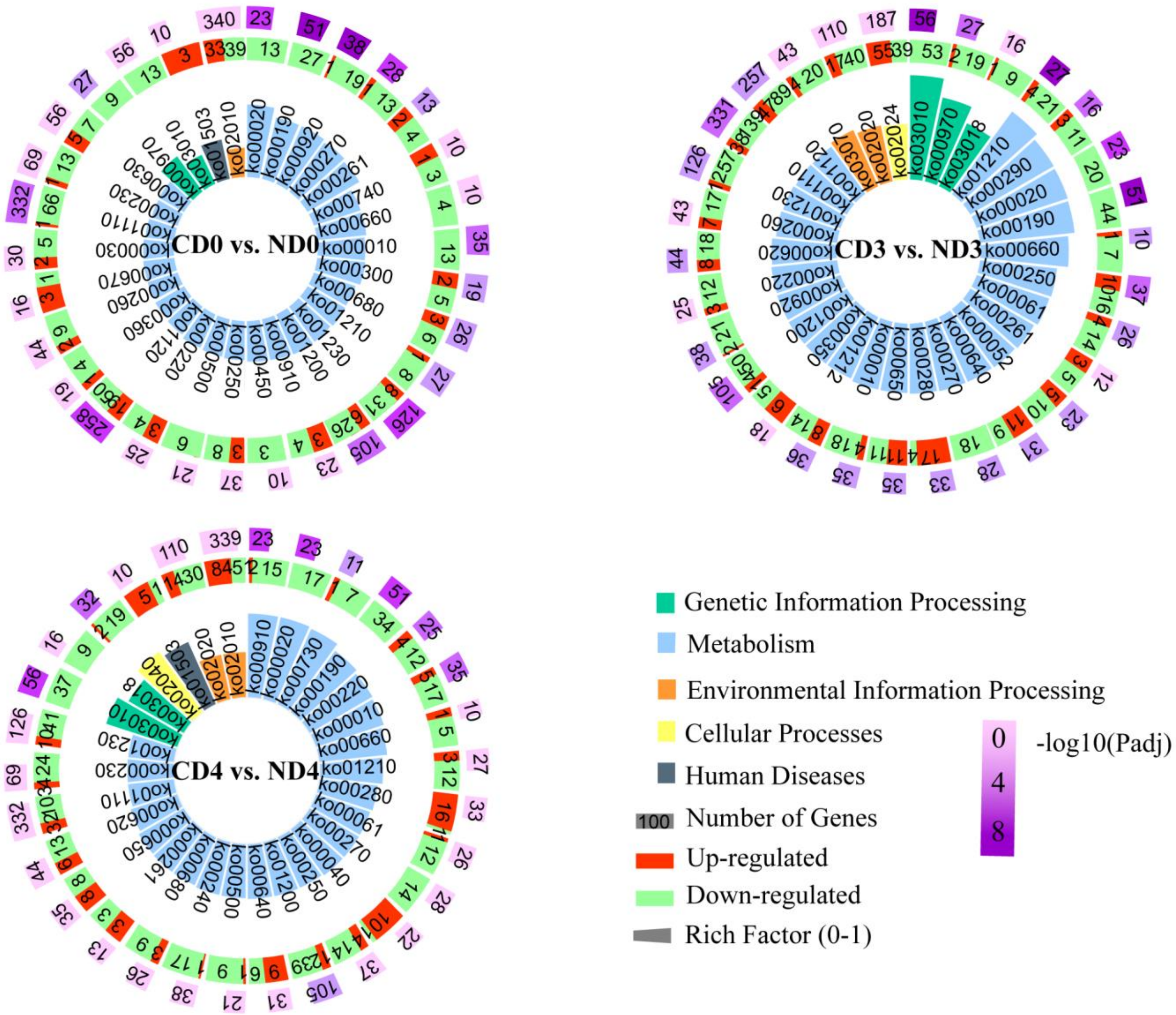

Figure S4 Kyoto Encyclopedia of Genes and Genome (KEGG) enrichment circle plots for the top 30 enriched pathways of three comparisons: CD0 (*Agrobacterium* cells co-cultivated with phylloplane of *Camellia sinensis* for 30 min) vs. ND0 (*Agrobacterium* cells co-cultivated with phylloplane of *N. benthamiana* for 30 min); CD3 (*Agrobacterium* cells co-cultivated with phylloplane of *C. sinensis* for 3 d) vs. ND3 (*Agrobacterium* cells co-cultivated with phylloplane of *N. benthamiana* for 3 d); CD4 (*Agrobacterium* cells co-cultivated with phylloplane of *C. sinensis* for 4 d) vs. ND4 (*Agrobacterium* cells co-cultivated with phylloplane of *N. benthamiana* for 4 d). There are three circles from the inside to the outside: the first circle is the pathway ID, the length of the bar indicate the rich factor (the number of DEGs/the number of background genes of this pathway), different colors represent different classifications; the second circle is the number of up-regulated genes and the down-regulated ones; the outside circle shows the number of the background genes and the adjusted p-value (P_{adj}) of the pathway.
