# Supplementary material for: Comparative Transcriptome Analysis of Agrobacterium tumefaciens Reveals the Molecular Basis for the Recalcitrant Genetic Transformation of Camellia sinensis L
Source: Biomolecules. 2022 May 11;12(5):688. doi: 10.3390/biom12050688 (PMC9138961; doi:10.3390/biom12050688)
Supplement: Supplementary file 1 [file biomolecules-12-00688-s001.zip › biomolecules-1645209-supplementary.zip!/Table S1.pdf]

Table S1 Primers used in qRT-PCR reaction.

Genes	Forward primers（5'-3'）	Reverse primers（5'-3'）
virE0	TGATGTTGATCGGACGGCTTTCG	ACACAGTGAGGTTGTCGGTTTGC
virA	TGCTGGCACACAAATCTACGGATC	GGCGGTGGCGAGTTCAAGAAG
upp	GCTGGCAATGGTCTTCTGGAAGG	GCCTCAAGCGTATCGTGGTCAC
flaB	AACTGGCTCGTCGGCGTCTC	GAGGGCGTATTTCGTGGTCGTAAC
flaA	CGAAGAGTCCACCAAGCTGAAGG	GCGGAAGAGCGACAGGATGTTC
ddpA	TTCCACTCCAACGACAAGTTCACG	GCGATATACTGGTTCCACGGGTTC
mcpV	AAGACGGGCGAATCCTTGAATACG	CCTGAATGCCGACCGACTGTTC
cheW2	AAGAGCACCGTCGCCAATGAAC	TGCCGGGATCGTGAGGATGTC
cheW1	TGTCCAACGCCATCAAGCAATCC	GTGTGCGGCATCGGGGTAAC
gyrB	CTCACCGACAAACGCCACTC	GCCGCCGTCATACAGCAA
Atu8171	TGGAAGAAGCGGAAATCA	AGCGAAACGACGGAGC
dnaC	GTTCCGACCTTATCGTGCTG	CGACGCCGCCATTCTT
