# Supplementary material for: Comparative Transcriptome Analysis of Agrobacterium tumefaciens Reveals the Molecular Basis for the Recalcitrant Genetic Transformation of Camellia sinensis L
Source: Biomolecules. 2022 May 11;12(5):688. doi: 10.3390/biom12050688 (PMC9138961; doi:10.3390/biom12050688)
Supplement: Supplementary file 1 [file biomolecules-12-00688-s001.zip › biomolecules-1645209-supplementary.zip!/Table S2.pdf]

**Table S2** RNA-seq results of mapping rate of raw reads against the reference genome of *Agrobacterium fabrum* str. C58 plus the plasmid pMKV060.

| Samples | Total reads | Total mapped | Multiple mapped | Uniquely mapped |
|---|---|---|---|---|
| CD0_1 | 7316074 | 7005294 (95.75%) | 72788 (0.99%) | 6932506 (94.76%) |
| CD0_2 | 7564976 | 7286516 (96.32%) | 70613 (0.93%) | 7215903 (95.39%) |
| CD0_3 | 7996972 | 7749459 (96.9%) | 71937 (0.9%) | 7677522 (96.01%) |
| ND0_1 | 6765696 | 6708287 (99.15%) | 92217 (1.36%) | 6616070 (97.79%) |
| ND0_2 | 6584706 | 6452072 (97.99%) | 80579 (1.22%) | 6371493 (96.76%) |
| ND0_3 | 7069846 | 6947639 (98.27%) | 95893 (1.36%) | 6851746 (96.92%) |
| CD3_1 | 7553458 | 7189164 (95.18%) | 67685 (0.9%) | 7121479 (94.28%) |
| CD3_2 | 7388542 | 7237515 (97.96%) | 38633 (0.52%) | 7198882 (97.43%) |
| CD3_3 | 7946468 | 7102322 (89.38%) | 60981 (0.77%) | 7041341 (88.61%) |
| ND3_1 | 7731228 | 7618172 (98.54%) | 112901 (1.46%) | 7505271 (97.08%) |
| ND3_2 | 8719236 | 8605288 (98.69%) | 165479 (1.9%) | 8439809 (96.8%) |
| CD4_1 | 7673658 | 7376618 (96.13%) | 60467 (0.79%) | 7316151 (95.34%) |
| CD4_2 | 8258274 | 8120056 (98.33%) | 73970 (0.9%) | 8046086 (97.43%) |
| CD4_3 | 7580880 | 7303322 (96.34%) | 49392 (0.65%) | 7253930 (95.69%) |
| ND4_1 | 8962786 | 8874886 (99.02%) | 112808 (1.26%) | 8762078 (97.76%) |
| ND4_2 | 7542930 | 7464069 (98.95%) | 121328 (1.61%) | 7342741 (97.35%) |
| ND4_3 | 8861532 | 8746602 (98.7%) | 111619 (1.26%) | 8634983 (97.44%) |
