# Supplementary material for: Comparative Transcriptome Analysis of Agrobacterium tumefaciens Reveals the Molecular Basis for the Recalcitrant Genetic Transformation of Camellia sinensis L
Source: Biomolecules. 2022 May 11;12(5):688. doi: 10.3390/biom12050688 (PMC9138961; doi:10.3390/biom12050688)
Supplement: Supplementary file 1 [file biomolecules-12-00688-s001.zip › biomolecules-1645209-tex.zip!/Definitions/biomolecules-1645209-g001.pdf]

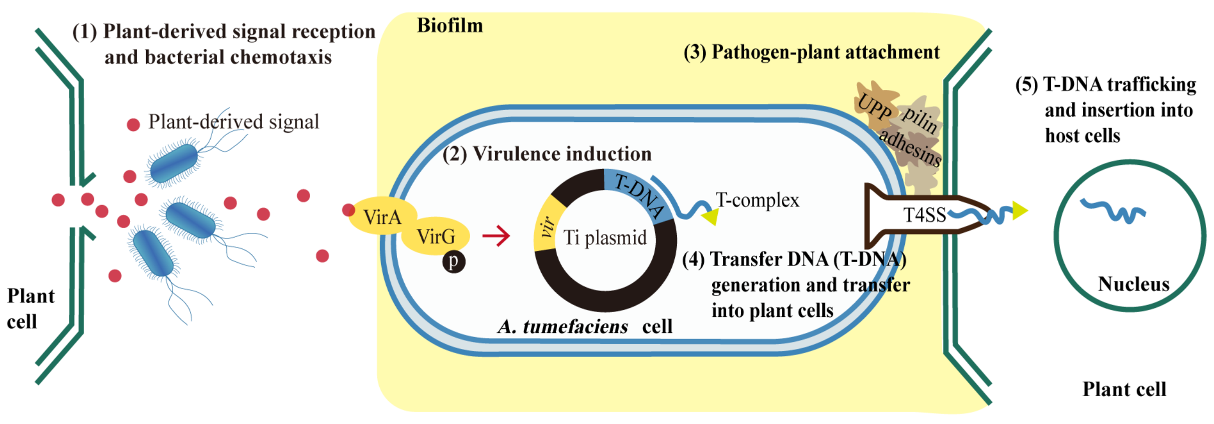
(1) Plant-derived signal reception and bacterial chemotaxis
Biofilm
(3) Pathogen-plant attachment
(5) T-DNA trafficking and insertion into host cells
Plant-derived signal
UPP
pilin
adhesins
(2) Virulence induction
T-DNA
T-complex
VirA
VirG
p
vir
Ti plasmid
T4SS
(4) Transfer DNA (T-DNA) generation and transfer into plant cells
Plant cell
A. tumefaciens cell
Nucleus
Plant cell
