# Supplementary material for: Comparative Transcriptome Analysis of Agrobacterium tumefaciens Reveals the Molecular Basis for the Recalcitrant Genetic Transformation of Camellia sinensis L
Source: Biomolecules. 2022 May 11;12(5):688. doi: 10.3390/biom12050688 (PMC9138961; doi:10.3390/biom12050688)
Supplement: Supplementary file 1 [file biomolecules-12-00688-s001.zip › biomolecules-1645209-tex.zip!/Definitions/biomolecules-1645209-g003.pdf]

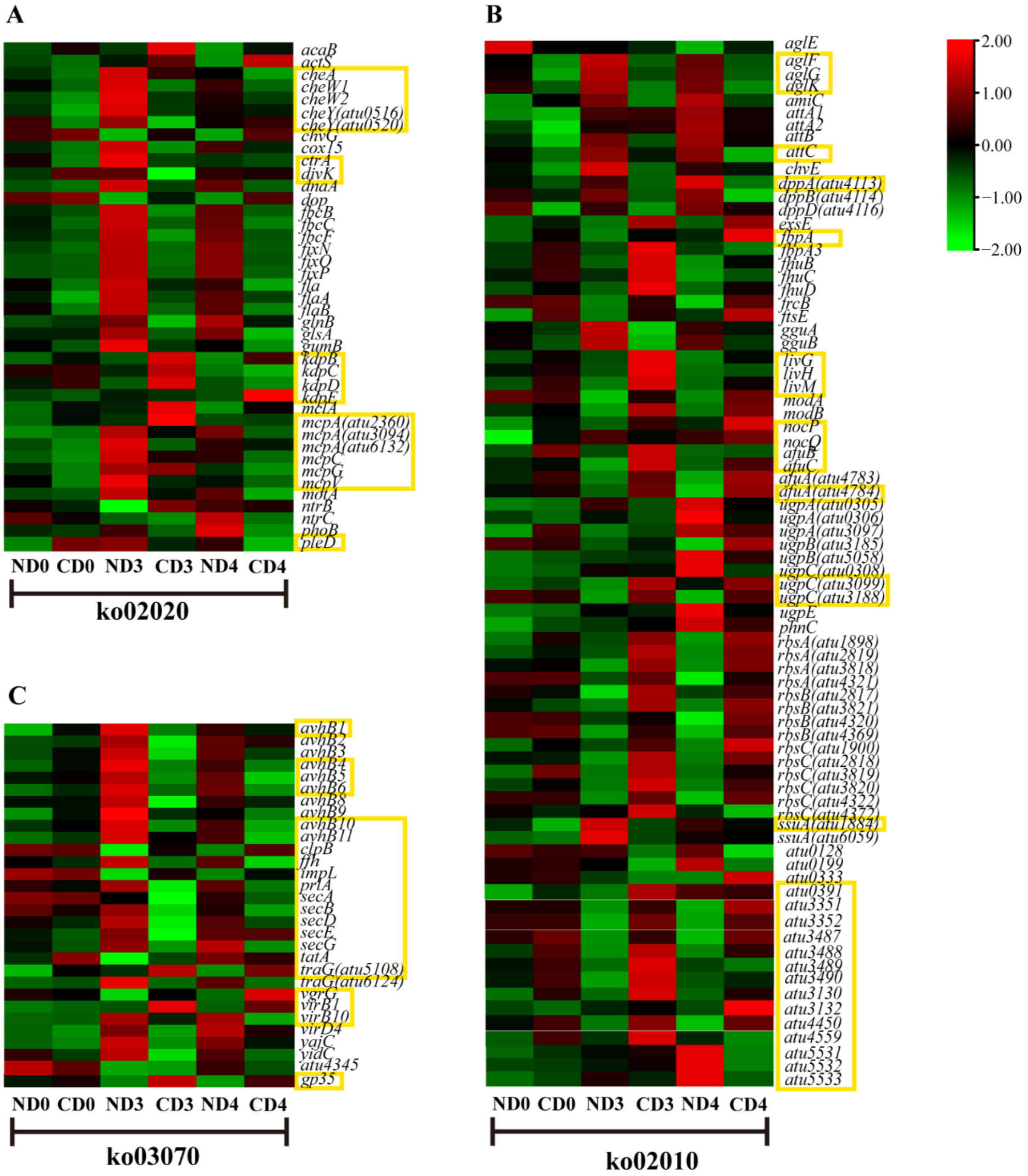
A
acaB
actS
cheA
cheW1
cheW2
cheY(atu0516)
cheY(atu0520)
chvG
cox15
ctrA
divK
dnaA
dop
fbcB
fbcC
fbcF
fixN
fixO
fixP
fla
flaA
flaB
glnB
glsA
gumB
kdpB
kdpC
kdpD
kdpE
mclA
mcpA(atu2360)
mcpA(atu3094)
mcpA(atu6132)
mcpC
mcpG
mcpV
motA
ntrB
ntrC
phoB
pleD
ND0 CD0 ND3 CD3 ND4 CD4
ko02020
B
aglE
aglF
aglG
aglK
amiC
attA1
attA2
attB
attC
chvE
dppA(atu4113)
dppB(atu4114)
dppD(atu4116)
exsE
fbpA
fbpA3
fhuB
fhuC
fhuD
frcB
ftsE
gguA
gguB
livG
livH
livM
modA
modB
nocP
nocQ
afuB
afuC
afuA(atu4783)
afuA(atu4784)
ugpA(atu0305)
ugpA(atu0306)
ugpA(atu3097)
ugpB(atu3185)
ugpB(atu5058)
ugpC(atu0308)
ugpC(atu3099)
ugpC(atu3188)
ugpE
phnC
rbsA(atu1898)
rbsA(atu2819)
rbsA(atu3818)
rbsA(atu4321)
rbsB(atu2817)
rbsB(atu3821)
rbsB(atu4320)
rbsB(atu4369)
rbsC(atu1900)
rbsC(atu2818)
rbsC(atu3819)
rbsC(atu3820)
rbsC(atu4322)
rbsC(atu4372)
ssuA(atu1884)
ssuA(atu6059)
atu0128
atu0199
atu0333
atu0391
atu3351
atu3352
atu3487
atu3488
atu3489
atu3490
atu3130
atu3132
atu4450
atu4559
atu5531
atu5532
atu5533
ND0 CD0 ND3 CD3 ND4 CD4
ko02010
2.00
1.00
0.00
−1.00
−2.00
C
avhB1
avhB2
avhB3
avhB4
avhB5
avhB6
avhB8
avhB9
avhB10
avhB11
clpB
ffh
impL
prlA
secA
secB
secD
secE
secG
tatA
traG(atu5108)
traG(atu6124)
vgrG
virB1
virB10
virD4
yajC
yidC
atu4345
gp35
ND0 CD0 ND3 CD3 ND4 CD4
ko03070
