# Supplementary material for: Comparative Transcriptome Analysis of Agrobacterium tumefaciens Reveals the Molecular Basis for the Recalcitrant Genetic Transformation of Camellia sinensis L
Source: Biomolecules. 2022 May 11;12(5):688. doi: 10.3390/biom12050688 (PMC9138961; doi:10.3390/biom12050688)
Supplement: Supplementary file 1 [file biomolecules-12-00688-s001.zip › biomolecules-1645209-tex.zip!/Definitions/biomolecules-1645209-g004.pdf]

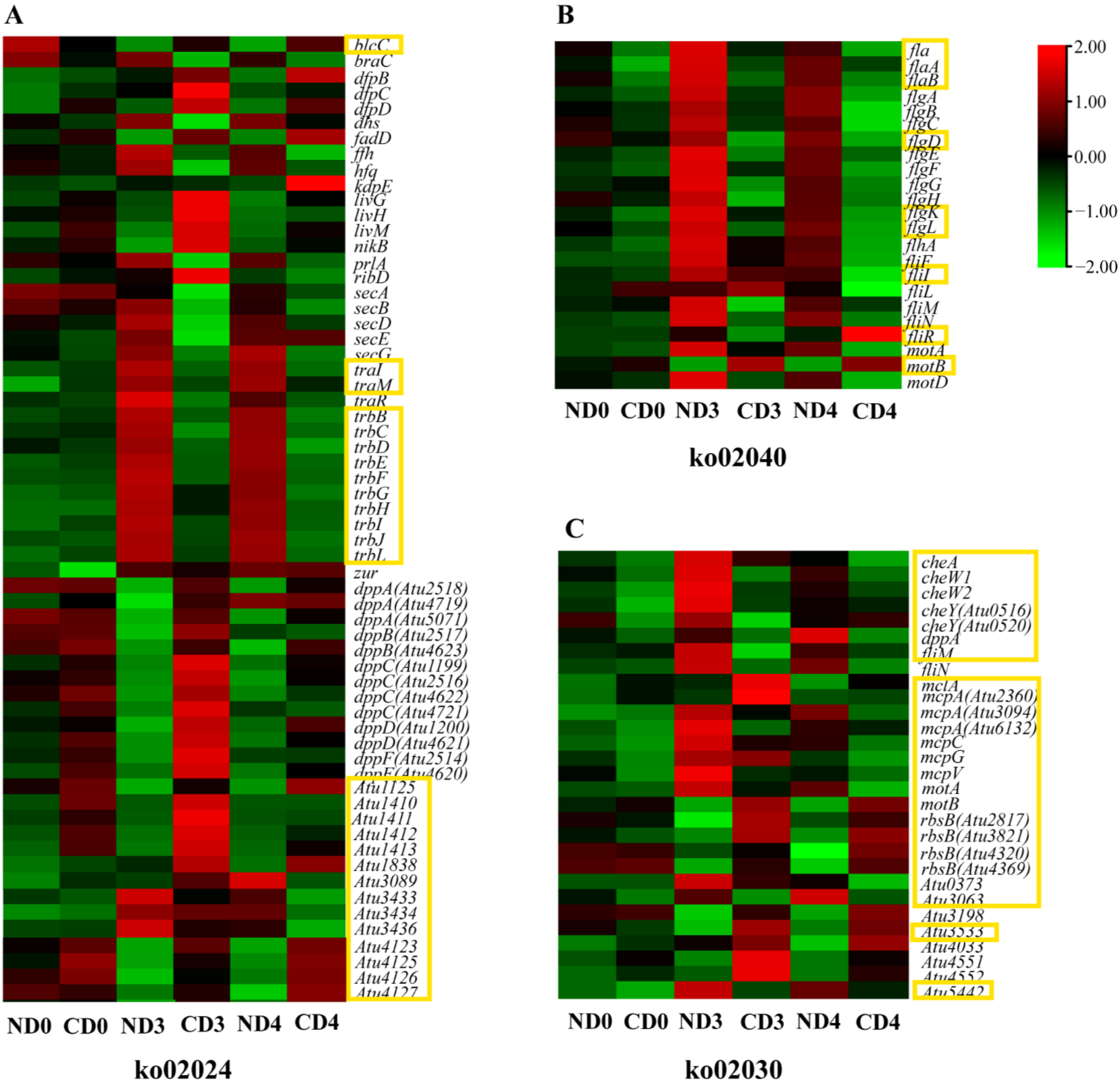
A
blcC
braC
dfpB
dfpC
dfpD
dhs
fadD
ffh
hfq
kdpE
livG
livH
livM
nikB
prlA
ribD
secA
secB
secD
secE
secG
traI
traM
traR
trbB
trbC
trbD
trbE
trbF
trbG
trbH
trbI
trbJ
trbL
zur
dppA(Atu2518)
dppA(Atu4719)
dppA(Atu5071)
dppB(Atu2517)
dppB(Atu4623)
dppC(Atu1199)
dppC(Atu2516)
dppC(Atu4622)
dppC(Atu4721)
dppD(Atu1200)
dppD(Atu4621)
dppF(Atu2514)
dppF(Atu4620)
Atu1125
Atu1410
Atu1411
Atu1412
Atu1413
Atu1838
Atu3089
Atu3433
Atu3434
Atu3436
Atu4123
Atu4125
Atu4126
Atu4127
ND0 CD0 ND3 CD3 ND4 CD4
ko02024
B
fla
flaA
flaB
flgA
flgB
flgC
flgD
flgE
flgF
flgG
flgH
flgK
flgL
flhA
fliF
fliI
fliL
fliM
fliN
fliR
motA
motB
motD
ND0 CD0 ND3 CD3 ND4 CD4
ko02040
2.00
1.00
0.00
−1.00
−2.00
C
cheA
cheW1
cheW2
cheY(Atu0516)
cheY(Atu0520)
dppA
fliM
fliN
mclA
mcpA(Atu2360)
mcpA(Atu3094)
mcpA(Atu6132)
mcpC
mcpG
mcpV
motA
motB
rbsB(Atu2817)
rbsB(Atu3821)
rbsB(Atu4320)
rbsB(Atu4369)
Atu0373
Atu3063
Atu3198
Atu3533
Atu4033
Atu4551
Atu4552
Atu5442
ND0 CD0 ND3 CD3 ND4 CD4
ko02030
