# Supplementary material for: Comparative Transcriptome Analysis of Agrobacterium tumefaciens Reveals the Molecular Basis for the Recalcitrant Genetic Transformation of Camellia sinensis L
Source: Biomolecules. 2022 May 11;12(5):688. doi: 10.3390/biom12050688 (PMC9138961; doi:10.3390/biom12050688)
Supplement: Supplementary file 1 [file biomolecules-12-00688-s001.zip › biomolecules-1645209-tex.zip!/Definitions/biomolecules-1645209-g005.pdf]

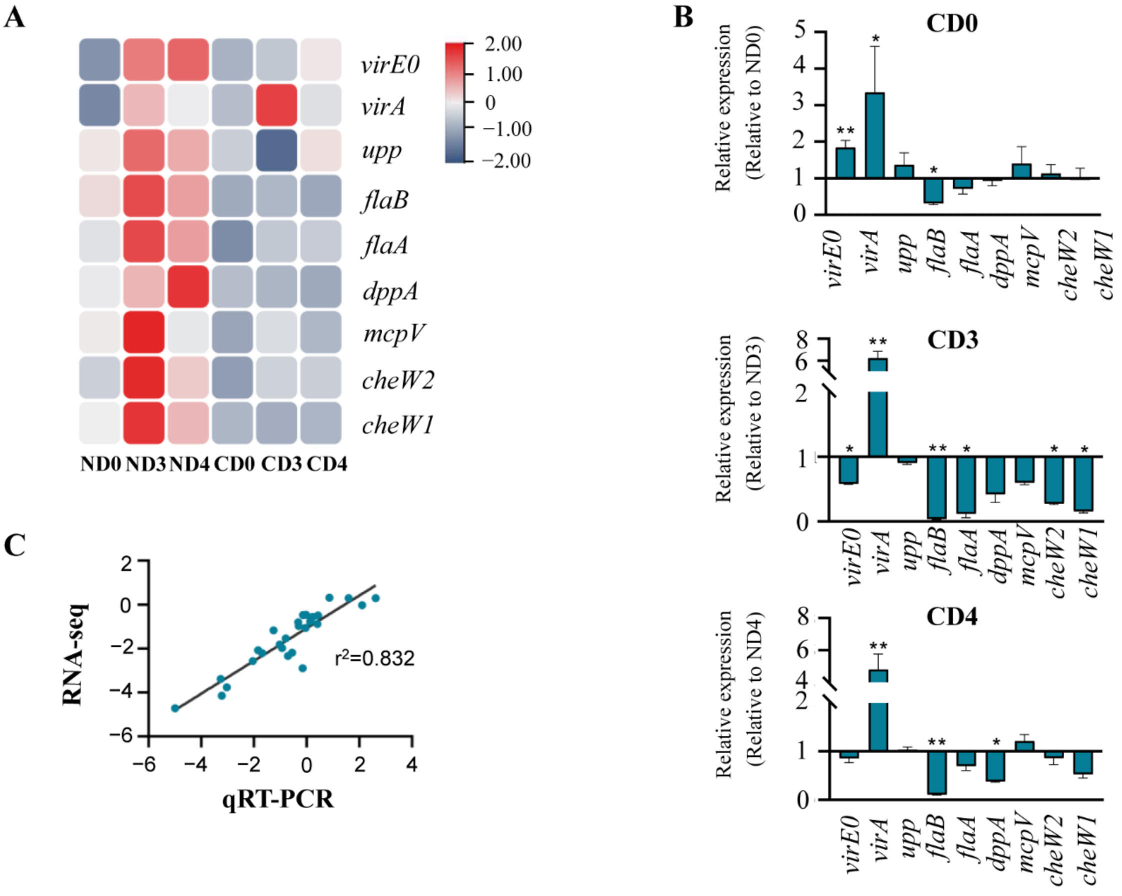
A
virE0
virA
upp
flaB
flaA
dppA
mcpV
cheW2
cheW1
ND0 ND3 ND4 CD0 CD3 CD4
2.00
1.00
0
−1.00
−2.00
B
CD0
Relative expression (Relative to ND0)
CD3
Relative expression (Relative to ND3)
CD4
Relative expression (Relative to ND4)
C
RNA-seq
qRT-PCR
r²=0.832
