# Supplementary material for: Comparative Transcriptome Analysis of Agrobacterium tumefaciens Reveals the Molecular Basis for the Recalcitrant Genetic Transformation of Camellia sinensis L
Source: Biomolecules. 2022 May 11;12(5):688. doi: 10.3390/biom12050688 (PMC9138961; doi:10.3390/biom12050688)
Supplement: Supplementary file 1 [file biomolecules-12-00688-s001.zip › biomolecules-1645209-tex.zip!/Definitions/biomolecules-1645209-g006.pdf]

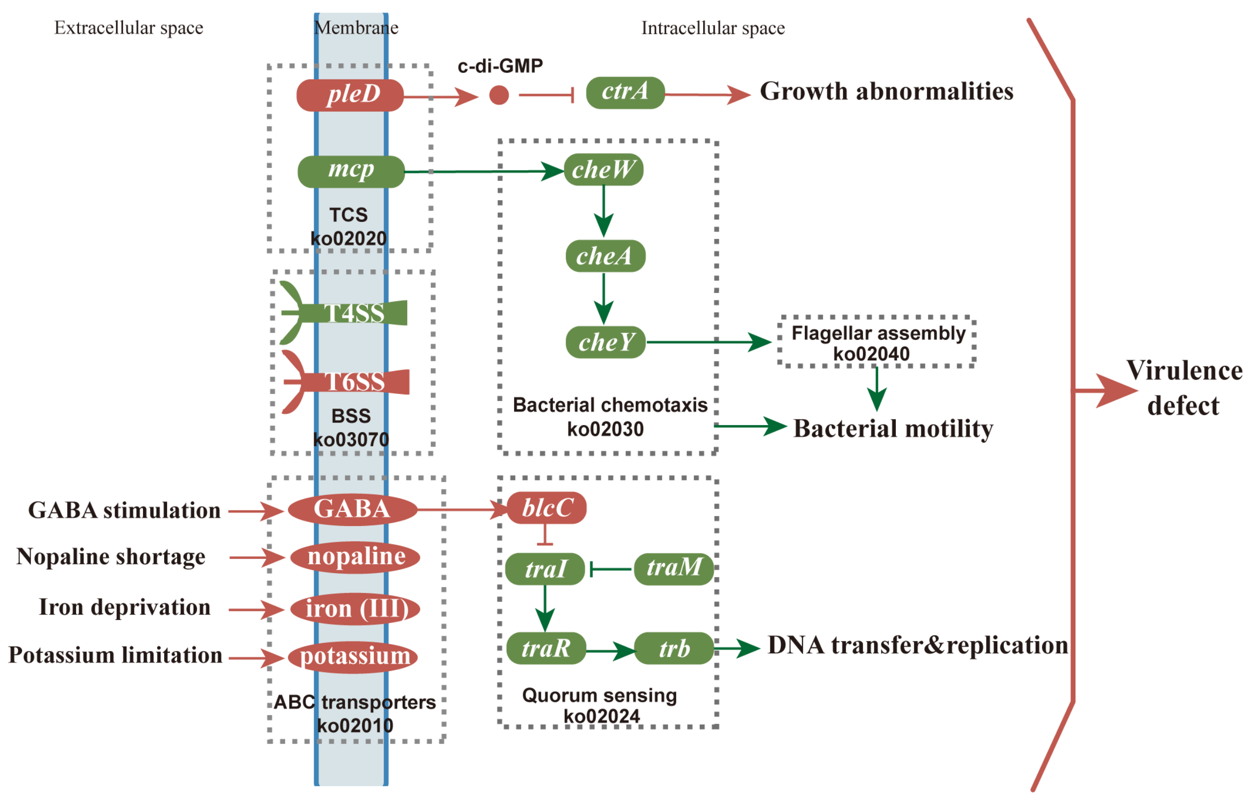
Extracellular space
Membrane
Intracellular space
c-di-GMP
pleD
ctrA
Growth abnormalities
mcp
cheW
cheA
cheY
TCS
ko02020
T4SS
T6SS
BSS
ko03070
Flagellar assembly
ko02040
Bacterial chemotaxis
ko02030
Bacterial motility
Virulence
defect
GABA stimulation
GABA
blcC
Nopaline shortage
nopaline
traI
traM
Iron deprivation
iron (III)
traR
trb
Potassium limitation
potassium
DNA transfer&replication
ABC transporters
ko02010
Quorum sensing
ko02024
