# Supplementary material for: Comparative Transcriptome Analysis of Agrobacterium tumefaciens Reveals the Molecular Basis for the Recalcitrant Genetic Transformation of Camellia sinensis L
Source: Biomolecules. 2022 May 11;12(5):688. doi: 10.3390/biom12050688 (PMC9138961; doi:10.3390/biom12050688)
Supplement: Supplementary file 1 [file biomolecules-12-00688-s001.zip › biomolecules-1645209-tex.zip!/Definitions/biomolecules-1645209-g007.pdf]

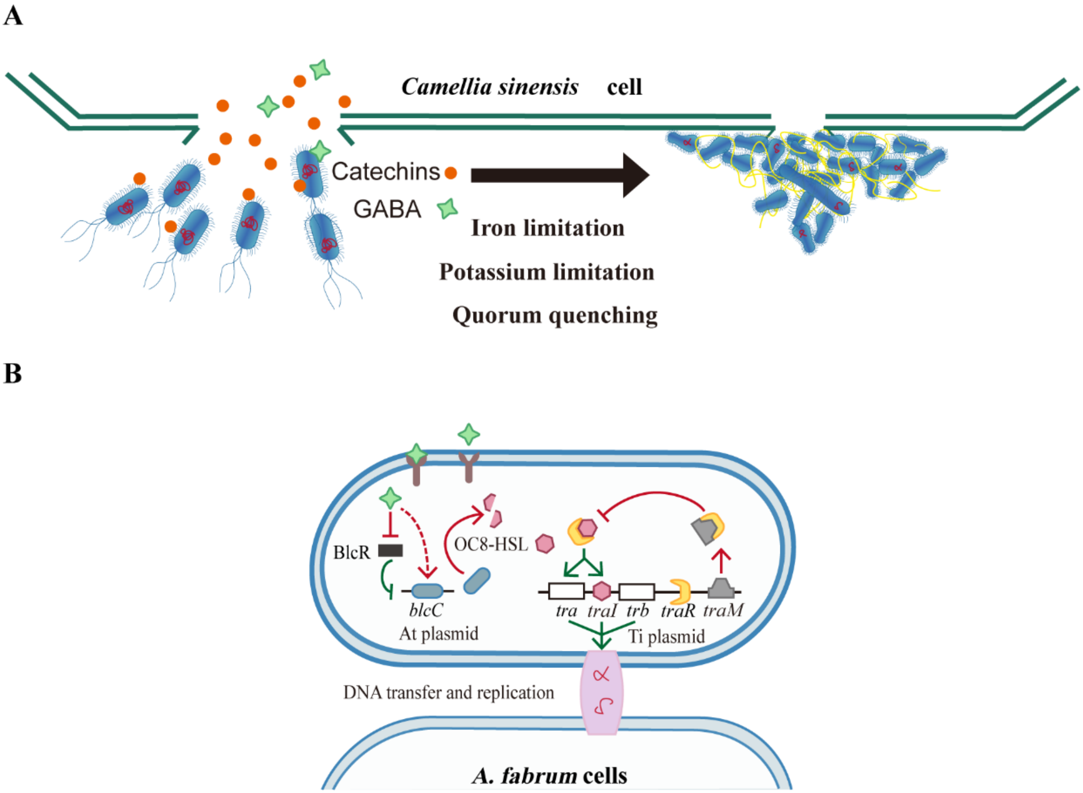
A
Camellia sinensis cell
Catechins
GABA
Iron limitation
Potassium limitation
Quorum quenching
B
BlcR
OC8-HSL
blcC
At plasmid
tra
traI
trb
traR
traM
Ti plasmid
DNA transfer and replication
A. fabrum cells
