# Supplementary material for: Comparative Transcriptome Analysis of Agrobacterium tumefaciens Reveals the Molecular Basis for the Recalcitrant Genetic Transformation of Camellia sinensis L
Source: Biomolecules. 2022 May 11;12(5):688. doi: 10.3390/biom12050688 (PMC9138961; doi:10.3390/biom12050688)
Supplement: Supplementary file 1 [file biomolecules-12-00688-s001.zip › biomolecules-1645209-tex.zip!/Definitions/logo-ccby-eps-converted-to.pdf]

cc BY
